# Risk factors for Alzheimer’s disease and cognitive function before middle age in a U.S. representative population-based study

**DOI:** 10.1016/j.lana.2025.101087

**Published:** 2025-04-05

**Authors:** Allison E. Aiello, Jennifer Momkus, Rebecca C. Stebbins, Yuan S. Zhang, Chantel L. Martin, Y. Claire Yang, Lauren Gaydosh, Taylor Hargrove, Adina Zeki Al Hazzouri, Kathleen Mullan Harris

**Affiliations:** a Robert N. Butler Columbia Aging Center, Columbia University, New York, NY, USA; b Department of Epidemiology, Mailman School of Public Health, Columbia University, New York, NY, USA; c Carolina Population Center, University of North Carolina at Chapel Hill, Chapel Hill, NC, USA; d Department of Epidemiology, University of North Carolina at Chapel Hill, Chapel Hill, NC, USA; e Department of Sociomedical Sciences, Mailman School of Public Health, Columbia University, New York, NY, USA; f Department of Sociology, University of North Carolina at Chapel Hill, Chapel Hill, NC, USA

**Keywords:** Alzheimer’s disease, Dementia risk factors, CAIDE risk score, Dementia risk score, APOE, Neurofilament light chain, Tau, ATN biomarkers, Immune biomarkers, Inflammatory biomarkers, Early midlife, Young adulthood, Life course, Cognitive function, US-Representative, Population-based

## Abstract

**Background:**

Alzheimer’s disease is a major health concern in the U.S., but most research has focused on older populations. We examined whether established risk factors and blood biomarkers are associated with cognition before midlife.

**Methods:**

Data from the National Longitudinal Study of Adolescent to Adult Health (Add Health) were analyzed. Participants were enrolled in 1994–95 (grades 7–12) and followed through 2018. We cross-sectionally analyzed weighted survey and biomarker data from Waves IV and V. We measured the Cardiovascular Risk Factors, Aging, and Incidence of Dementia (CAIDE) score comprised of age, education, sex, systolic blood pressure, body mass index, cholesteroal, and physical activity and apolipoprotein E ε4 allele (APOE ε4) status. We also measured total Tau and Neurofilament light (NfL), high sensitivity C-reactive protein (hsCRP), Interleukin (IL)-1β, IL-6, IL-8, IL-10, and Tumor necrosis factor alpha (TNF-α). Outcomes included immediate word recall, delayed word recall, and backward digit span.

**Findings:**

Analytic sample sizes ranged from 4507 to 11,449 participants in Wave IV and from 529 to 1121 participants in Wave V. The survey-weighted median (IQR) age was 28 (26–29) years in Wave IV and 38 (36–29) years in Wave V. About half of the survey-weighted Wave IV participants were female (48.4–52.1% across analytic samples), 71.4–72.5% were White, 12.5–14.9% were Black, and 9.3–10.2% were Hispanic. In Wave V, 43.6–46.8% were female, 68.7–69.3% were White, 17.1%–20.0% were Black, and 7.3%–9.6% were Hispanic. The CAIDE score was associated with all cognition measures in Wave IV. For example, among adults aged 24–34, each 1-point increase in CAIDE was associated with a 0.03 standard deviation lower backward digit span score (95% CI: −0.04, −0.02). Total Tau was associated with immediate word recall in Wave V (β = −0.13, 95% CI: −0.23, −0.04). Wave IV hsCRP and IL-10 and Wave V IL-6, IL-1β, and IL-8 were also associated with lower cognitive scores.

**Interpretation:**

Key risk factors for Alzheimer’s Disease are linked to cognitive function as early as ages 24–44, highlighting the need for early prevention in the US.

**Funding:**

10.13039/100000002NIHP01HD31921, U01AG071448, U01AG071450, R01AG057800, P30AG066615, T32HD091058, P2CHD050924.

**Research in context**

**Evidence before this study**

The majority of existing research on risk factors for Alzheimer’s Disease has focused on study samples of individuals 50 years of age and older. There is a dearth of research on risk factors for Alzheimer’s Disease prior to midlife. This presents a significant gap in our understanding of when risk factors emerge and begin to associate with outcomes related to the clinical onset of Alzheimer’s Disease, such as cognitive function. Therefore, we undertook a comprehensive search of PubMed and Google Scholar between January 1st, 2020 and January 1st, 2025 to identify studies examining key risk factors for Alzheimer’s disease —including risk scores, apolipoprotein E (APOE), immune/inflammatory biomarkers, and amyloid, tau, and neurodegenerative biomarkers—prior to middle age, with no limitations on publication dates. The search terms we used included combinations of “Alzheimer’s Disease (AD) risk factors”, “dementia risk factors”, “APOE”, “Neurofilament light chain”, “total Tau”, “Amyloid Tau Neurodegenerative (ATN) biomarkers”, “immune biomarkers”, “inflammatory biomarkers”, “CAIDE risk score”, “dementia risk score”, or “AD risk score”, AND “midlife”, “early midlife”, “young adulthood”, or “life course”, AND “cognitive function”, AND “US-representative”, or “population-based”, with no language restrictions, focusing on studies reported associations between Alzheimer’s Disease risk factors and cognitive function in younger populations. We reviewed studies based on the age range of participants, prioritizing those as close as possible to younger or early-middle-aged individuals, as no studies specifically focused on the age group central to our research. Notably, none of the reviewed studies examined all the risk factors in a single publication as assessed here, and we found no population-representative studies with a comparable age range.

**Added value of this study**

Our study is the first to systematically examine Alzheimer’s Disease risk factors in a large sample of generally healthy individuals within a representative U.S. cohort of 24- to 44-year-olds. We specifically focused on this age range, identifying the earliest associations between key Alzheimer’s Disease risk factors and cognitive function patterns in the U.S. population before midlife. Our findings suggest that risk profiles for Alzheimer’s Disease emerge before middle age, revealing early manifestations of cognitive patterns that may predict the decline typically observed in older populations. Additionally, we found that certain Alzheimer’s Disease risk factors, including cardiovascular, ATN, and immune biomarkers, are prevalent in individuals before middle age and may play a role in shaping early cognitive function. Conversely, APOE ε4 status showed no significant early association with cognition in this age group, aligning with prior research suggesting that APOE ε4 is less predictive of cognitive outcomes in younger populations.

**Implications of all the available evidence**

Our large, population-representative cohort of pre-middle-aged participants underscores the importance of identifying Alzheimer’s Disease risk factors decades before most available neurology studies typically begin to focus. Our results emphasize the need for early monitoring of emerging risk factors, including cardiovascular, immune, and ATN risks, as targets for Alzheimer’s Disease prevention. While our findings align with literature focusing on middle and later life, we observed notable distinctions, including the lack of association with a significant genetic risk factor and TNF-alpha, which has shown inconsistent links to Alzheimer’s Disease in previous research. The potential impact of our findings is significant, providing clinicians and health researchers with a clearer understanding of the early emergence of Alzheimer’s Disease risk factors before middle age and laying the groundwork for earlier detection and prevention approaches.

## Introduction

Alzheimer’s disease poses a global health challenge.^1^ While most research in the United States (US) has focused on older populations, where the risk of dementia is highest, there is a lack of studies investigating risk factors for Alzheimer’s Disease and cognitive function in young to early midlife adults before the onset of disease. Identifying pathways to Alzheimer’s Disease and cognitive impairment before older age is critical for slowing the expected growth of Alzheimer’s Disease in coming decades.

The landscape of risk factors that are known to shape Alzheimer’s Disease risk in older age encompass social, behavioral, clinical, and biological dimensions.^1^ On the social and behavioral side, factors such as lower educational attainment, physical inactivity, and smoking are notable risk factors. Clinically, health conditions such as diabetes, obesity, hypertension, and high cholesterol are identified as consistent contributors to Alzheimer’s Disease risk. In recent years, researchers have sought to combine the social, behavioral, and clinical risk factors into comprehensive risk scores for Alzheimer’s Disease.^2^ The Cardiovascular Risk Factors, Aging, and Incidence of Dementia (CAIDE) score is among the most extensively researched risk algorithms and integrates social, behavioral, and biological risks including education, sex, age, cholesterol levels, blood pressure, body mass index, and physical activity.^3^

On the biological side, genetic, neurological, immune, and inflammatory molecules have been implicated as biomarkers of Alzheimer’s Disease risk.^4^ The genetic profile of apolipoprotein E (APOE) is a known risk factor for Alzheimer’s Disease in older populations.^5^ Moreover, neuropathological markers, such as amyloid beta and neurofibrillary tangles, have been investigated as predictors of Alzheimer’s Disease in later life. The amyloid (A), tau (T), and neurodegeneration (N) (known as ATN) biomarkers are increasingly promising for predicting Alzheimer’s Disease risk in older populations.^6^ Two commonly measured biomarkers are total tau and neurofilament light chain (NfL). Both measures are predictive of later life Alzheimer’s Disease risk in older populations.^7^^,^^8^ Furthermore, central and peripheral immune and inflammatory mechanisms are increasingly recognized as pivotal in the pathogenesis of Alzheimer’s Disease. A recent comprehensive review identified relatively consistent positive associations between several inflammation-related cytokines and Alzheimer’s Disease risk, with the two most-studied markers being interleukin-6 (IL-6) and tumor necrosis factor-alpha (TNF-α).^4^ While also widely studied, C-reactive protein was less consistently associated with Alzheimer’s Disease risk in this review, with approximately 25% of the studies demonstrating positive associations, 25% showing negative associations, and roughly 50% showing no significant association. Less well-studied candidate immune and inflammatory markers have been examined in relation to Alzheimer’s Disease, such as IL-1β and IL-8, but the small body of existing studies showed inconsistent findings.^4^

While most studies analyzing CAIDE risk scores, ATN, and immune biomarkers have focused on older populations, their relationship with cognitive function in pre-midlife adults has rarely been investigated. This study aims to address several significant gaps in our understanding of Alzheimer’s Disease risk in the US population by examining widely studied risk factors for Alzheimer’s Disease and cognitive function before midlife. This is crucial because many have hypothesized a long subclinical phase of Alzheimer’s Disease but have rarely studied Alzheimer’s Disease markers in early adulthood. Specifically, we assessed the associations between CAIDE score, APOE ε4 status, two ATN biomarkers (total Tau and NfL), and several inflammatory molecules and interleukins (highly sensitive C- Reactive Protein (hsCRP), IL-1β, IL-6, IL-8, IL-10, TNF-α) with three standard measures of cognitive function in a US representative study of 24- to 44-year-old participants in the National Longitudinal Study of Adolescent to Adult Health (Add Health).^9^

## Methods

Study Population: Data from Waves IV-V of the National Longitudinal Study of Adolescent to Adult Health (Add Health) were used for this study and analyzed cross-sectionally at each wave. Add Health is a nationally representative cohort tracking adolescent participants since 1994–1995 (Wave I) through four follow-up waves.^9^

Wave IV (n = 15,701), conducted in 2008, when participants were 24–34 years old, included in-home interviews, cognitive tests, physical exams, saliva collection, and dried blood spot collection from all eligible respondents. Wave V, conducted between 2016 and 2018 when participants were aged 34–44, was a mixed-mode survey combining in-person and web/mail surveys with a target of 12,300 survey participants, out of which 1702 received in-home interviews. Cognitive tasks were administered in-person by field interviewers during the in-home interviews. All Wave V participants were invited to complete a separate “biovisit”, including a physical exam and venous blood collection, resulting in visits arranged for 5381 participants. Additional information on study design, response rates, sample collection, and testing procedures are available at Harris et al., 2019 and on the Add Health website.^9^^,^^10^ Participants who reported being pregnant were removed (Wave V samples N = 7, Wave IV CAIDE N = 455, Wave IV Blood-Based N = 361) from analyses except those involving APOE status.

### Measures

#### CAIDE score

The CAIDE score is a weighted composite score based on established risk factors for dementia. There are two versions; both include age, education, biological sex, systolic blood pressure (SBP), body mass index (BMI), total cholesterol, and physical activity. Version 2 adds APOE ε4 status. The weights are the coefficients derived from the original study, creating the CAIDE score.^3^ We aimed to align as closely as possible with the variables used in the original CAIDE study while adapting to our specific sample and available data. A summary of the components and scoring are shown in Table 1. We examined both CAIDE version 1 and version 2.

**Table 1:** Cardiovascular Risk Factors, Aging, and Incidence of Dementia (CAIDE) Score Components.

CAIDE Risk Factors	Version 1 Weighted Components	Version 2 Weighted Components
Age	Lowest tertile = 0 Middle tertile = 3 Highest tertile = 4	Lowest tertile = 0 Middle tertile = 3 Highest tertile = 5
Educational attainment	College degree or higher = 0 Some college/technical training = 2 HS diploma/GED or less = 3	College degree or higher = 0 Some college/technical training = 3 HS diploma/GED or less = 4
Biological sex	Female = 0 Male = 1	Female = 0 Male = 1
Systolic blood pressure	≤140 mm Hg = 0 >140 mm Hg = 2	≤140 mm Hg = 0 >140 mm Hg = 2
BMI	≤30 kg/m2 = 0 >30 kg/m2 = 2	≤30 kg/m2 = 0 >30 kg/m2 = 2
Total cholesterol	Bottom 9 deciles = 0 Top decile = 2	Bottom 9 deciles = 0 Top decile = 1
Physical activity	≥ activity 2× per week = 0 < activity 2× per week = 1	≥ activity 2× per week = 0 < activity 2× per week = 1
APOE ε4 status	N/A	Non-ε4 carrier = 0 ≤1 ε4 allele = 2

APOE = Apolipoprotein E.

#### APOE ε4

A total of N = 11,550 participants had available genomic data. The Michigan Imputation Server was used for imputation on unphased data. Data were imputed using 1000 Genomes Project phase 3 version 5.^11^ Using the imputed rs7412 (R^2^ score = 0.896) and rs429358 (R^2^ score = 0.919) variants, we categorized participants as ε2/ε2, ε2/ε3, ε2/ε4, ε3/ε3, ε3/ε4, and ε4/ε4. APOE status was defined by having at least one ε4 allele (i.e., those with APOE ε2/ε4, ε3/ε4, or ε4/ε4 phenotypes vs. ε2/ε2, ε3/ε3, or ε2/ε3).

#### Amyloid, Tau, and Neurodegeneration (ATN) biomarkers

Among those who completed a separate biovisit in Wave V, N = 4940 venous blood samples were collected via phlebotomy. Using a digital enzyme-linked immunosorbent assay (ELISA), serum samples were assayed for NfL, and plasma samples for total Tau.

#### Immune biomarkers

Among Wave IV respondents, 75.9% consented to contribute a dried blood spot sample and to sample archival (N = 11,917). Dried blood spot samples were assayed using a high-sensitivity ELISA for CRP (hsCRP) in dried blood spot samples^12^ and subsequently archived. A subset of archived dried blood spot samples with sufficient quantity (N = 5019) were later tested for additional inflammatory markers, including interleukins (IL-6, IL-8, and IL-10) and TNF-α, using a highly sensitive electrochemiluminescent immunoassay (ECLIA) modified for dried blood spot samples.^13^ In Wave V, serum samples from the biovisit (N = 4940) were assayed for hsCRP immediately upon receipt at the testing laboratory using a hsCRP-specific particle-enhanced immunonephelometric assay. Archived samples were later tested for inflammatory cytokines (IL-6, IL-10, IL-8, IL-1β, and TNF- α) using an electrochemiluminescence immunoassay.^14^ Units for all blood biomarkers are in pg/mL except for hsCRP (mg/L). Values were log-transformed then standardized.

#### Cognitive function

Home interviewers conducted cognitive assessments among all participants in Wave IV (N = 14,732) and those who received an in-home interview in Wave V (N = 1717). In each wave, three cognitive assessments included immediate word recall, delayed word recall, and backward digit span. Participants were read a 15-word list for word recall and asked to recall as many of the 15 words as possible. Cognitive scores were standardized into a z-score for analysis, with a higher score indicating higher memory cognition.

#### Covariates

We identified covariates for inclusion based on Directed Acyclic Graphs (DAG) models.^15^ Early life economic status was operationalized using the social origins score, based on parental education, occupation, household income, and receipt of public assistance.^16^ A higher score suggests higher economic status in adolescence. Additional covariates include age at exam, educational attainment (at the same wave in which the biomarker was measured), sex assigned at birth, smoking status (never vs. past vs. current), and an indicator for recent inflammatory conditions. A race/ethnicity variable was constructed from the Wave V survey and supplemented with Wave I data if missing. Participants self-selected one or more boxes from a list including: “American Indian or Alaska Native”, “Asian”, “Black, African American”, “Hispanic”, “Pacific Islander”, “White”, and “Some other race or origin”. Please see Supplementary Methods (Section A) for more information on variables and conceptualization of race and ethnicity.

#### Analysis

The independent variable, dependent variable, and sample size of each analytic sample are shown in Table 2. Flow charts illustrating the selection of each sample are provided in the supplementary material (Supplementary Figs. S1 and S2). Survey-weighted descriptive statistics were compiled to characterize demographics and relevant study variables in each sample. To examine the cross-sectional associations between cardiovascular (e.g., CAIDE score), ATN, immune, and genetic risk with cognitive function, survey-weighted linear regressions were used to estimate β parameters for unadjusted and adjusted models with each independent variable predicting each participants cognitive score in the same wave. For the CAIDE score models, covariates included race/ethnicity categories, social origins score, and an indicator for inflammatory conditions. For the ATN and immune blood based biomarker models, covariates included sex assigned at birth, race/ethnicity categories, educational attainment, age, and an indicator for recent inflammatory conditions. For the APOE status, covariates included age and sex assigned at birth. We also conducted several sensitivity analyses to assess: 1) whether results were similar among individuals who have the same set of variables available at both Wave IV and Wave V (see Supplementary Fig. S3), 2) sample selection biases (see Supplementary Figs. S4–S7), 3) alternative groupings of APOE ε4 status (see Supplementary Figs. S8 and S9), and 4) longitudinal assessment of Wave V cognition adjusting for Wave IV in a subset of participants with available data across both waves (see Supplementary Methods, Section C and Supplementary Figs. S10 and S11).

**Table 2:** Analytic samples.

Wave	Sample	Independent variable	Dependent variable	Sample size	Results shown
IV	CAIDE	CAIDE score	Cognitive scores	N = 11,449	Fig. 1
V	CAIDE	CAIDE score	Cognitive scores	N = 529	Fig. 1
IV	Genetic	APOE status	Cognitive scores	N = 10,674	Fig. 2
V	Genetic	APOE status	Cognitive scores	N = 1121	Fig. 2
IV	Blood-Based Biomarker	Immune risk biomarkers	Cognitive scores	N = 4507	Fig. 4
V	Blood-Based Biomarker	ATN biomarkers	Cognitive scores	N = 588	Fig. 3
V	Blood-Based Biomarker	Immune risk biomarkers	Cognitive scores	N = 588	Fig. 4

Wave IV was conducted in 2008 when participants had a median age of 28 years (IQR: 36-29 years), Wave V was conducted in 2016–2018 when participants had a median age of 38 (IQR: 36–39 years).

CAIDE = Cardiovascular Risk Factors, Aging, and Incidence of Dementia, ATN = Amyloid (A), Tau (T), and Neurodegeneration (N), APOE Status = Apolipoprotein E, defined as having at least one ε4 allele (i.e., those with APOE ε2/ε4, ε3/ε4, or ε4/ε4 phenotypes vs. ε2/ε2, ε3/ε3, or ε2/ε3).

The study was conducted in compliance with University of North Carolina at Chapel Hill Institutional Review Board (Add Health Wave V: IRB #13–1946, initial approval 05/08/2013). In addition, we received Columbia University IRB approval for the analysis (Add Health Wave V: IRB AAAU4491, initial approval: 12/20/2022). Further, Add Heath participants gave written informed consent for participation in all aspects of this study in accordance with the University of North Carolina Institutional Review Board guidelines.

#### Role of the funding source

The funders had no role in study design, data collection, data analysis, interpretation, or writing of the report.

## Results

The weighted characteristics of each analytic sample are shown in Table 3, and a comparison with the overall Add Health sample is available in Supplementary Table S2. In Wave IV, the analytic sample sizes ranged from N = 4507 for the immune risk factors to N = 11,449 for the CAIDE score, with a median age of 28 years (IQR: 26–29 years). In the Wave IV CAIDE, Blood-Based, and Genetic samples respectively, the weighted percentage of female participants was 48.4% (N = 5984), 52.1% (N = 2451), and 50.0% (N = 5704). The weighted percentage with a college degree or higher was 30.3% (N = 3665) in the Wave IV CAIDE sample, 26.4% (N = 1268) in the Wave IV Blood-Based sample, and 29.9% (N = 3354) in the Wave IV Genetic Sample. The weighted distribution of race and ethnicity in the Wave IV CAIDE sample was 71.4% White (N = 6798), 14.1% Black, African American (N = 2262), and 10.1% Hispanic (N = 1530). The Wave IV Blood-Based sample was 72.5% White (N = 2700), 12.5% Black, African American (N = 815), and 10.2% Hispanic (N = 625). The Wave IV Genetic sample was 72.0% White (N = 6470), 14.9% Black, African American (N = 2151), and 9.3% Hispanic (N = 1361). In the Wave V CAIDE (N = 529), Blood-Based (N = 588), and Genetic (N = 1121) samples respectively, the weighted percentage of female participants was 43.8% (N = 277), 43.6% (N = 208), and 46.8% (N = 581). The weighted percentage with a college degree or higher was 36.1% (N = 208) in the Wave V CAIDE sample, 33.4% (N = 222) in the Wave V Blood-Based sample, and 30.7% (N = 375) in the Wave V Genetic Sample. The weighted distribution of race and ethnicity in the Wave V CAIDE sample was 69.3% White (N = 338), 20.0% Black, African American (N = 104), and 7.3% Hispanic (N = 59). The Wave V Blood-Based sample was 68.7% White (N = 366), 19.5% Black, African American (N = 117), and 8.5% Hispanic (N = 74). The Wave IV Genetic sample was 69.2% White (N = 686), 17.1% Black, African American (N = 231), and 9.6% Hispanic (N = 138).

**Table 3:** Weighted sample characteristics, National Longitudinal Study of Adolescent to Adult Health (Add Health) Waves IV–V.

	Wave IV CAIDE sample	Wave V CAIDE sample	Wave IV Blood-Based Biomarker sample	Wave V Blood-Based Biomarker sample	Wave IV genetic sample	Wave V genetic sample
	n = 11,449	n = 529	n = 4507	n = 588	n = 10,674	n = 1121
Age (years)	27.8 (26.3, 29.3)	38.4 (36.7, 39.7)	27.8 (26.3, 29.3)	38.2 (36.7, 39.7)	27.8 (26.3, 29.3)	37.9 (36.5, 39.4)
Sex						
Female	5984, 48.4%	277, 43.8%	2451, 52.1%	308, 43.6%	5704, 50.0%	581, 46.8%
Race						
American Indian or Alaska Native	116, 1.0%	–	42, 0.8%	–	79, 0.7%	–
Asian	653, 2.9%	20, 2.4%	287, 3.5%	20, 2.1%	528, 2.5%	43, 2.5%
Black, African American	2262, 14.1%	104, 20.0%	815, 12.5%	117, 19.5%	2151, 14.9%	231, 17.1%
Hispanic	1530, 10.1%	59, 7.3%	624, 10.2%	74, 8.5%	1361, 9.3%	138, 9.6%
Pacific Islander	55, 0.3%	–	26, 0.3%	–	53, 0.3%	–
Some other race or origin	35, 0.2%	–	13, 0.2%	–	32, 0.3%	–
White	6798, 71.4%	338, 69.3%	2700, 72.5%	366, 68.7%	6470, 72.0%	686, 69.2%
Education						
College Degree or Higher	3665, 30.3%	208, 36.1%	1268, 26.4%	222, 33.4%	3354, 29.9%	375, 30.7%
Some College and/or Technical Training	5125, 43.7%	220, 44.1%	2106, 46.0%	248, 44.7%	4776, 43.4%	499, 44.8%
High School/GED or lower	2659, 26.0%	101, 19.8%	1133, 27.6%	118, 21.9%	2544, 26.6%	247, 24.4%
Recent Inflammatory Condition^a^	1788, 16.0%	82, 16.8%	747, 16.9%	95, 17.3%	1673, 15.9%	99, 9.6%
Social Origins Score^b^	0.1 (−0.7, 1.0)	0.3 (−0.6, 1.1)	0.1 (−0.7, 0.9)	0.2 (−0.7, 1.1)	0.2 (−0.7, 1.0)	0.1 (−0.8, 1.0)
CAIDE score v1^c^	5.2 (3.2, 7.2)	6.1 (3.9, 8.0)	5.4 (3.3, 7.3)	6.2 (3.9, 8.1)	5.3 (2.5, 4.8)	6.1 (3.8, 7.9)
CAIDE score v2^c^ (with APOE status)	6.7 (4.3, 9.1)	7.7 (5.3, 9.9)	7.0 (4.5, 9.3)	7.7 (5.3, 10.0)	6.8 (4.3, 9.1)	7.6 (5.1, 9.8)
*missing*	2764	9	795	41	1128	568
Systolic blood pressure > 140 mm Hg	1382, 12.8%	80, 15.7%	560, 14.4%	88, 16.6%	1230, 12.3%	96, 16.8%
*missing*	0	0	123	23	272	519
BMI > 30 kg/m2	4191, 36.6%	253, 50.1%	1720, 38.6%	288, 51.4%	3911, 37.0%	310, 52.1%
*missing*	0	0	53	8	131	504
Total Cholesterol ≥ 90th percentile	1107, 10.1%	49, 9.7%	507, 12.1%	53, 9.1%	1030, 10.8%	55, 8.7%
*missing*	0	0	5	5	807	541
Physical activity < 2× per week	2554, 21.8%	96, 19.2%	985, 21.7%	113, 19.4%	2424, 22.3%	225, 20.5%
*missing*	0	0	4	0	5	0
Smoking status						
Never smoker	5827, 46.8%	267, 46.1%	2111, 43.1%	300, 47.9%	5450, 47.3%	560, 46.1%
Ever smoker	1321, 12.6%	100, 20.9%	516, 11.8%	109, 19.2%	1286, 12.9%	209, 20.8%
Current smoker	4301, 40.6%	162, 33.1%	1880, 45.2%	179, 32.9%	3938, 39.8%	352, 33.0%
*missing*	0	0	0	0	0	0
Blood-based Biomarkers (log-transformed)						
hsCRP (mg/L)	0.7 (−0.3, 1.6)	0.6 (−0.3, 1.5)	0.8 (−0.2, 1.7)	0.7 (−0.2, 1.6)	0.8 (−0.2, 1.7)	0.7 (−0.2, 1.7)
TNF-α (pg/mL)	1.1 (0.9, 1.3)	0.9 (0.7, 1.5)	1.1 (0.9, 1.3)	0.9 (0.7, 1.1)	1.1 (0.9, 1.3)	0.9 (0.7, 1.1)
IL-6 (pg/mL)	−0.2 (−0.6, 0.3)	−0.3 (−0.8, 0.1)	−0.2 (−0.6, 0.3)	−0.3 (−0.7, 0.2)	−0.2 (−0.6, 0.3)	−0.3 (−0.7, 0.2)
IL-10 (pg/mL)	−0.9 (−1.4, −0.4)	−1.5 (−1.8, −1.1)	−0.9 (−1.4, −0.4)	−1.5 (−1.8, −1.1)	−0.9 (−1.4, −0.4)	−1.5 (−1.8, −1.1)
IL-8 (pg/mL)	4.3 (4.0, 4.6)	2.6 (2.3, 3.0)	4.3 (4.0, 4.6)	2.6 (2.3, 3.0)	4.3 (4.0, 4.6)	2.6 (2.3, 3.0)
IL-1B (pg/mL)	NA	−3.7 (−5.0, −2.9)	NA	−3.7 (−5.0, −2.9)	NA	−3.7 (−5.0, −2.9)
NfL (pg/mL)	NA	1.9 (1.6, 2.2)	NA	1.9 (1.6, 2.2)	NA	1.9 (1.6, 2.2)
Total Tau (pg/mL)	NA	0.8 (0.5, 0.8)	NA	0.8 (0.4, 1.1)	NA	0.8 (0.4, 1.1)
APOE ε4 status						
ε4 carrier (ε2/ε4, ε3/ε4, or ε4/ε4)	2368, 27.3%	154, 29.1%	1043, 27.0%	169, 27.6%	2943, 27.4%	315, 27.6%
*missing*	2764	9	659	9	0	0
Cognitive function tests						
Immediate word recall	6.0 (4.8, 7.4)	5.8 (4.5, 7.1)	6.1 (4.8, 7.5)	5.6 (4.4, 7.0)	6.0 (4.8, 7.4)	5.6 (4.3, 6.9)
Delayed word recall	4.6 (3.3, 5.9)	4.3 (2.9, 5.6)	4.7 (3.4, 6.0)	4.1 (2.7, 5.4)	4.6 (3.3, 6.0)	4.1 (2.8, 5.4)
Backwards digit span	3.5 (2.5, 4.8)	3.6 (2.4, 5.1)	3.6 (2.5, 4.9)	3.5 (2.4, 5.0)	3.5 (2.5, 4.8)	3.5 (2.4, 4.9)

Data are shown as N, survey-weighted % for categorical variables and weighted median, (IQR) for continuous variables.

Wave IV was conducted in 2008 when participants had a median age of 28 years (IQR: 26–29 years), Wave V was conducted in 2016–2018 when participants had a median age of 38 (IQR: 36–39 years).

CAIDE = Cardiovascular Risk Factors, Aging, and Incidence of Dementia, BMI = Body Mass Index, hsCRP = high sensitivity C-Reactive Protein, TNF-α = Tumor Necrosis Factor alpha, IL = Interleukin, NfL = Neurofilament Light, APOE = Apolipoprotein E.

a Indicator of any of the following conditions: gum disease, active infection, injury, acute illness, and/or surgery in the past 4 weeks, and/or fever in the past 2 weeks. Reported use of cox-2 inhibitors, corticotrophines, glucocorticords, anti-rheumatics, anti-psoriatics, immunosuppressive agents or monoclonal antibodies in the past 4 weeks included in Wave V.

b Factor score based on Wave I parental reports of education, occupation, household income, and household receipt of public assistance (standardized to a z-score for analysis).^12^

c CAIDE score version 1 is a weighted sum of education, sex, age, total cholesterol, systolic blood pressure, body mass index, and physical activity. Version 2 includes the same variables and adds APOE ε4 carrier status.

**Fig. 1:**
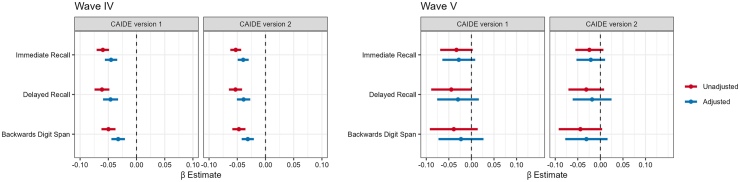
**Association between CardiovascularRisk Factors,Aging, and Incidence of Dementia (CAIDE) Score andCognitive Test Scores, National LongitudinalStudy of Adolescence to AdultHealth (Add Health)Wave IV andWave V.** Wave IV was conducted in 2008 when participants had a median age of 28 years (IQR: 26–29 years), Wave V was conducted in 2016–2018 when participants had a median age of 38 (IQR: 36–39 years). APOE = Apolipoprotein E, APOE status defined as having at least one ε4 allele (i.e., those with APOE ε2/ε4, ε3/ε4, or ε4/ε4 phenotypes vs. ε2/ε2, ε3/ε3, or ε2/ε3). CAIDE score version 1 (v1) is a weighted sum of education, sex, age, total cholesterol, systolic blood pressure, body mass index, and physical activity. Version 2 (v2) includes the same variables and adds APOE status. Each panel shows the β estimates and 95% CIs for survey-weighted linear regressions where CAIDE scores are the independent variable and cognitive function tests scores are the dependent variable. Models are adjusted for race/ethnicity, social origin score, an indicator for recent inflammatory conditions, and smoking status. Wave IV: N = 11,449 for the CAIDE version 1 score, N = 8685 for the CAIDE version 2 score (with APOE e4 carrier status). Wave V: N = 529 for CAIDE score version 1, N = 520 for CAIDE score version 2.

CAIDE scores. In Wave IV, higher CAIDE scores (both versions) were statistically significantly associated with lower cognitive scores in all domains (Fig. 1 and Supplementary Table S3). At Wave V, the effect estimates for the CAIDE scores were lower in magnitude and not statistically significant in fully adjusted models (Fig. 1 and Supplementary Table S3). In supplementary analyses (see Supplementary section C), we explored associations between CAIDE scores and cognitive measures across participants who had data on all variables and covariates in both Wave IV and Wave V (Supplementary Fig. S3 and Table S4, N = 412). Similar patterns were observed for both Waves IV and V, but some results included the null value in the 95% confidence intervals after the reductions in sample size.

### Apolipoprotein (APOE) ε4

**Fig. 2:**
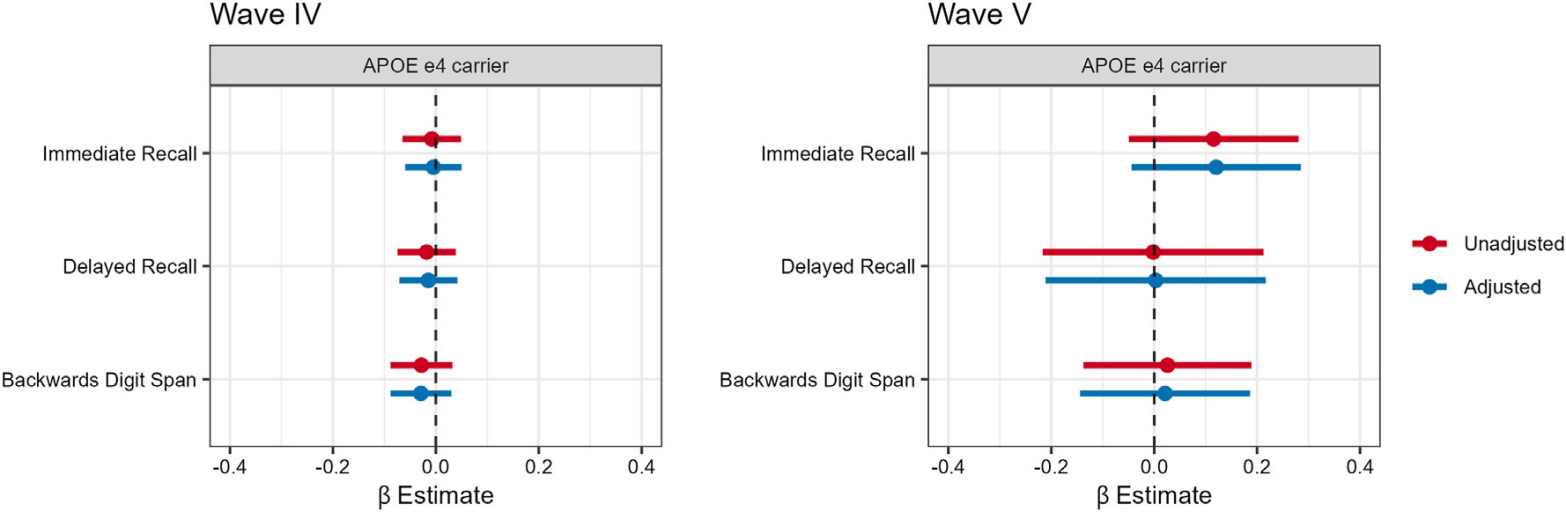
**Association between APOE Status and Cognitive Function, National LongitudinalStudy of Adolescent toAdultHealth (AddHealth)Wave IV andWave V.** Wave IV was conducted in 2008 when participants had a median age of 28 years (IQR: 26–29 years), Wave V was conducted in 2016–2018 when participants had a median age of 38 (IQR: 36–39 years). APOE = Apolipoprotein E, APOE status defined as having at least one ε4 allele (i.e., those with APOE ε2/ε4, ε3/ε4, or ε4/ε4 phenotypes vs. ε2/ε2, ε3/ε3, or ε2/ε3). Each panel shows the β estimates and 95% CIs for survey-weighted linear regressions where APOE e4 carrier status (carrier vs. non-carrier) is the independent variable and cognitive function tests scores are the dependent variable. Models adjusted for sex assigned at birth and age. Wave IV N = 10,674, Wave V N = 1121.

There were no associations between having at least one ε4 allele and cognitive function in Wave IV nor Wave V (Fig. 2 and Supplementary Table S3). We also examined the same associations among participants with data on all variables and covariates in both Wave IV and V (Supplementary Fig. S3 and Table S4, N = 1063). In this subsample, there were similarly no associations between APOE status and cognitive function.

### ATN markers

**Fig. 3:**
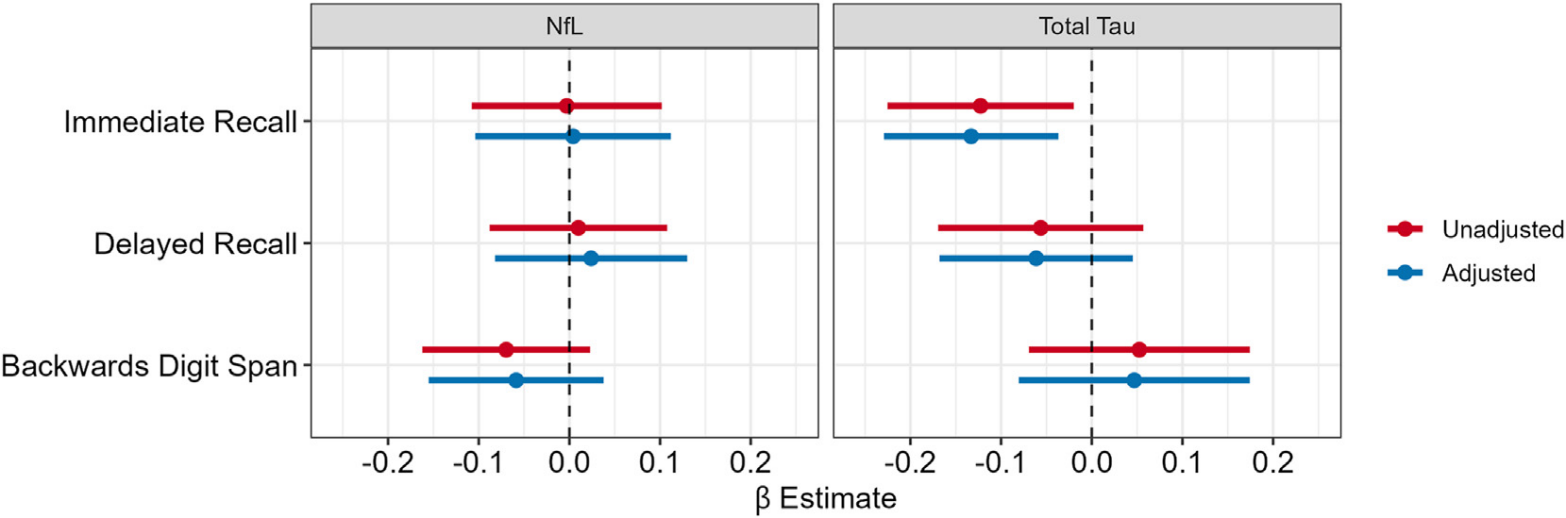
**Association between Amyloid, Tau, and Neurodegeneration (ATN) biomarkers and cognitive function,National Longitudinal Study of Adolescent to Adult Health (Add Health) Wave V, N = 588.** Add Health Wave V was conducted in 2016–2018 when participants had a median age of 38 (IQR: 36–39 years). NfL = Neurofilament Light. Each panel shows the β estimate and 95% CI for survey-weighted linear regressions where Wave V blood-based ATN biomarker concentrations are the independent variable and Wave V cognitive function tests scores are the dependent variable. Models adjusted for race/ethnicity, education, sex assigned at birth, age, an indicator for inflammatory conditions, and smoking status. NfL N = 588, total Tau N = 584.

There was a negative association between total Tau and immediate word recall at Wave V shown in Fig. 3 and Supplementary Table S3 (β = −0.13, 95% CI: −0.23, −0.04), but not with delayed recall nor the backward digit span. Nfl was associated with lower backward digit span scores, though it was not statistically significant (β = −0.06, 95% CI: −0.16, 0.04). There were no statistically significant associations between Nfl and the other cognitive measures.

### Immune risk factors

**Fig. 4:**
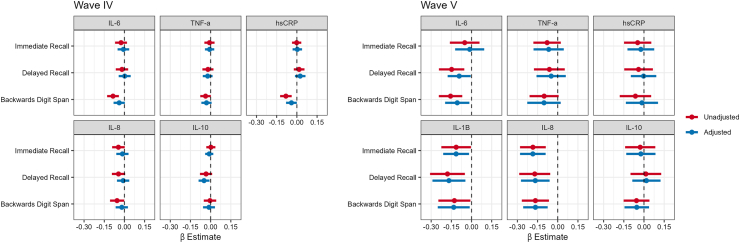
**Association betweenImmuneRisk Biomarkers and CognitiveTest Scores,National Longitudinal Study of Adolescent to AdultHealth (AddHealth) Wave IV andWave V.** Wave IV was conducted in 2008 when participants had a median age of 28 years (IQR: 26–29 years), Wave V was conducted in 2016–2018 when participants had a median age of 38 (IQR: 36–39 years). IL = Interleukin, TNF-α = Tumor Necrosis Factor alpha, hsCRP = high sensitivity C-Reactive Protein. Each panel shows the β estimates and 95% CIs for cross-sectional survey-weighted linear regressions where immune risk biomarker concentrations are the independent variable and cognitive function tests scores are the dependent variable. Wave IV cross-sectional associations are on the left and Wave V cross-sectional associations are on the right. Models adjusted for race/ethnicity, educational attainment, sex assigned at birth, age, an indicator for inflammatory conditions, and smoking status. Wave IV (dried blood spots): N = 4485 for hsCRP, N = 4507 for all other biomarkers. Wave V (serum): N = 567 for hsCRP, N = 588 for all other biomarkers.

Cross-sectional associations between each inflammatory biomarker and cognitive scores are shown in Fig. 4 β estimates are also shown in Supplementary Table S3. In Wave IV, IL-6 and hsCRP were associated with lower backwards digit span scores, but the estimate for IL-6 was not statistically significant after adjusting for covariates (IL-6: β = −0.04 SDs, 95% CI: −0.08, 0.00, hsCRP: β = −0.04, 95% CI: −0.08, −0.001). IL-10 was associated with delayed recall scores (β = −0.05, 95% CI: −0.09, −0.01). None of the other biomarkers (TNF-α, IL-8) were associated with lower cognitive scores in Wave IV (Supplementary Table S3). In Wave V, IL-6 was associated with lower backward digit span (β = −0.10, 95% CI: −0.19, −0.01) and delayed recall (β = −0.09, 95% CI: −0.18, −0.01), and IL-8 was associated with lower cognitive scores in all domains (immediate recall: β = −0.19, 95% CI: −0.28, −0.09; delayed recall: β = −0.17, 95% CI: −0.27, −0.06; backward digit span: β = −0.17, 95% CI: −0.26, −0.08), even after adjustment for covariates. IL-1β was associated with lower immediate (β = −0.15, 95% CI: −0.28, −0.03) and delayed recall scores (β = −0.12, 95% CI: −0.21, −0.02), and lower backward digit span (β = −0.13, 95% CI: −0.25, −0.01). See Supplementary Table S3 in the Supplementary material for β estimates and 95% CIs of all the associations shown in the figures.

## Discussion

Our findings shed light on the emergence of Alzheimer’s Disease risk factors in early adulthood in the U.S. population over the forthcoming two to four decades. Several key risk factors for Alzheimer’s Disease were associated with standard measures of cognition in 24- to 44-year-olds in the U.S., suggesting that these factors may be related to cognitive function decades before the onset of Alzheimer’s Disease. The CAIDE score was consistently linked to all three cognitive function measures (immediate recall, delayed recall, and backward digit span). Notably, a key genetic risk factor, APOE ε4, was not associated with recall nor backward digit span, suggesting that the effects of APOE ε4 may not become apparent until middle to older age. We also observed significant associations with ATN biomarkers. Specifically, total Tau was associated with lower immediate word recall but not backward digit span scores. Although higher levels of NfL showed a trend toward lower backward digit span scores, the association was not statistically significant. A limited number of immune markers were significantly associated with cognitive function at ages 24–34 years; however, some of these associations appeared to become more robust in the following decade of life. For instance, IL-6 was not associated with backward digit span in Wave IV but showed a significant association with lower backward digit span in Wave V. Similarly, IL-8 was not associated with cognitive scores in Wave IV but was associated with all three measures of cognition in Wave V. These findings suggest that cardiovascular, ATN, and immune markers may be associated with critical measures of cognitive function at much younger ages than previously recognized, with some associations becoming more prominent between the early midlife ages of 33 and 44.

We observed associations between the CAIDE score and all three measures of cognition in Wave IV. For example, among adults aged 24–34, each 1-point increase in the CAIDE score was associated with a 0.03 SD decrease in the average backward digit span score, adjusted for covariates (95% CI: −0.04, −0.02). Among adults aged 34–44, each 1-point increase in the CAIDE score was similarly associated with a 0.03 SD decrease in the average backward digit span score, adjusted for covariates (95% CI: −0.08, 0.02). The magnitudes of these associations were consistent across waves for each cognitive test. Still, they did not reach statistical significance in Wave V. It is possible that sample size in Wave V was too small to detect statistically significant associations. Prior research supports robust associations between the CAIDE score and cognitive function in older cohorts, although these relationships varied depending on the cognitive domain and study population. For instance, studies in a US cohort (mean age 60.1 years) and a Finnish cohort (mean age 52.4 years) found consistent associations between higher CAIDE scores at baseline and lower subsequent scores in all domains tested (including memory tasks).^17^^,^^18^ In a New Zealand cohort, researchers identified an inverse association between the CAIDE score and midlife intelligence quotient and subjective midlife cognitive problems at age 45.^2^ However, a study in Northern Manhattan (mean age 64 years) found cross-sectional inverse associations between the CAIDE score and tests of executive function and processing speed, but no association with memory tests or decline in test scores over time.^19^ Finally, a study in the UK (mean age 56 years) showed associations between the CAIDE score and declines in reasoning, vocabulary, and global cognitive scores but not in memory scores.^20^ Importantly, all these studies focused on midlife or older ages (i.e., the average ages in each sample were above 45 years at baseline). To the best of our knowledge, no studies have examined the relationship between the CAIDE score and cognition in young adulthood and early midlife in the U.S. Our study contributes to this body of knowledge by suggesting that these significant associations are observable well before age 50.

In contrast to prior research on APOE ε4 and cognitive function in older populations, we did not observe significant associations between the APOE ε4 allele (heterozygous nor homozygous) and our measures of cognitive function. Our findings are consistent with previous research showing that the impact of APOE ε4 is not evident in younger populations.^21^^,^^22^ Taken together, these results support the hypothesis that tau-related pathology in conjunction with APOE ε4 may either accumulate gradually after age 50 or activate more rapidly in older age due to other factors.^21^, ^22^, ^23^ Future research tracking these associations in longitudinal studies, such as Add Health, may help uncover the critical temporal changes in cognition related to APOE ε4.

Of note, two key ATN biomarkers appear to be associated with different aspects of memory at ages 34–44. NfL was associated, though not statistically significantly, with lower backward digit span scores, a measure of working memory. In contrast, total Tau was significantly associated with poorer immediate word recall, a measure of verbal episodic memory. Remarkably, these patterns are consistent with research on these markers in much older populations at risk of Alzheimer’s Disease. For example, NfL, a marker of neuronal injury, is particularly associated with working memory and processing speed.^24^ A study involving older adults from the National Health and Nutrition Examination Survey (NHANES) reported an inverse relationship between serum NfL levels and digit symbol substitution test (DSST) scores in older participants.^24^ Our finding of an association between Tau levels and episodic memory is noteworthy because Tau has been shown to be cross-sectionally associated with worse episodic memory, particularly among older individuals, including those without significant cognitive impairment.^25^, ^26^, ^27^ Together, these findings provide preliminary evidence that ATN biomarkers may influence episodic and working memory a decade before middle age. To our knowledge, these are the first findings to suggest that ATN biomarkers are associated with cognition in a relatively large sample of 34–44-year-olds. Future research is warranted to examine these and other promising ATN biomarkers in younger individuals.

We observed that several immune markers were associated with cognitive measures at ages 24–34, with IL-6 and hsCRP associated with backward digit span. In the next decade of life, IL-6 continued to be associated with backward digit span, while IL-1β and IL-8 appeared to be associated with word recall and backward digit span in Wave V but not Wave IV. In contrast, we found no associations with hsCRP in Wave V. Chronic systemic inflammation, often measured by elevated levels of inflammatory biomarkers like CRP and IL-6, has been linked to an increased risk of developing Alzheimer’s Disease.^28^ A recent review by Li et al. found that IL-6 was the most consistently associated biomarker with Alzheimer’s Disease, and IL-1β was also consistently positively associated with Alzheimer’s Disease.^4^ However, these studies were primarily conducted in populations over the age of 50 years. Our findings suggest the potential emergence of IL-6 and IL-1β as important immune molecules related to cognition early in the life course, consistent with this review of later-life relationships. Our evidence for these immune and inflammatory relationships as early as the third and fourth decades of life, as observed in this study, is noteworthy and suggests that associations between these markers and cognition are apparent 10–20 years earlier than what prevailing theories suggest.^4^

This study has several limitations. The significant associations observed between CRP and IL-10 in Wave IV were not present in Wave V. This may be due to differences in sample size, where the larger sample in Wave IV allowed for the detection of statistically significant but smaller effect sizes, whereas in Wave V, effect estimates were closer to the null. Alternatively, these differences may reflect true variability in biomarker-cognition relationships across waves. The limited sample size of the longitudinal data, precluded us from examining trends over time and ATN biomarkers were only available in Wave V. Wave IV samples were assayed for cytokines using blood spots, while Wave V samples were assayed using serum or plasma. As previously reported, IL-8 in particular, showed lower agreement between blood spot and serum assays.^13^ This discrepancy might explain why we observed an association between IL-8 and cognition in Wave V but not in Wave IV, which used blood spot assays. While there is a possibility of type I errors due to testing multiple models, we did not correct for multiple comparisons because each model was designed to address distinct research questions that were determined *a priori*. Nonetheless, this study is groundbreaking because it is the first to examine key Alzheimer’s Disease risk factors in a large diverse population-based sample of 24- to 44-year-olds in the US.

In conclusion, our study highlights that established risk factors and biomarkers implicated in Alzheimer’s Disease, including cardiovascular, ATN, and immune markers, are associated with cognitive function much earlier in life than previously recognized, with significant associations observed as early as ages 24–44. The CAIDE score, in particular, consistently predicted cognitive scores across multiple domains in young adults, while APOE ε4’s impact may not emerge until later in life. ATN and immune and inflammatory biomarkers are already beginning to show associations with cognition pre-midlife. Our findings suggest that key risk factors for Alzheimer’s Disease in older age, begin to shape cognitive function decades before clinically detectable cognitive impairment and Alzheimer’s Disease, underscoring the importance of early prevention across the life course.

## Contributors

Allison E. Aiello–conceptualization, data curation, methodology, funding acquisition, supervision, writing-original draft, writing-review & editing, decision to submit for publication.

Jennifer Momkus–formal analysis, visualization, methodology, validation, writing-original draft, writing-review and editing.

Rebecca C. Stebbins–methodology, writing-original draft, writing-review & editing.

Yuan S. Zhang–formal analysis, writing-review & editing.

Chantel L. Martin–writing-review & editing, funding acquisition.

Y. Claire Yang–writing-review & editing, funding acquisition.

Lauren Gaydosh–writing-review & editing, funding acquisition.

Taylor Hargrove–writing-review & editing, funding acquisition.

Adina Zeki Al Hazzouri–writing-review & editing.

Kathleen Mullan Harris–conceptualization, data curation, methodology, supervision, writing-review & editing, funding acquisition.

## Data sharing statement

All data used to in the present study, including individual de-identified participant data, and a data dictionary, will be available to Add Health Users in 2026. Public-use data are available from three sources: Add Health Dataverse hosted by UNC’s Research Data Management Core, the Inter-university Consortium for Political and Social Research (ICPSR), and the Association of Religion Data Archives (ARDA). Users may obtain the data from any of these sources, depending on their needs.

During the final editing of this work the author(s) used Grammarly, inc (2025) in order to provide suggested edits to the manuscript for grammar, wording, and brevity. After using this tool/service, the author(s) reviewed and edited the content as needed and take(s) full responsibility for the content of the publication.

## Declaration of interests

The authors have no conflict’s of interest to declare.
