# Supplementary material for: Risk factors for Alzheimer’s disease and cognitive function before middle age in a U.S. representative population-based study
Source: Lancet Reg Health Am. 2025 Apr 5;45:101087. doi: 10.1016/j.lana.2025.101087 (PMC12001091; doi:10.1016/j.lana.2025.101087)
Supplement: Supplementary material [file mmc1.docx]

**Title: Risk Factors for Alzheimer's Disease and Cognitive Function Before Middle Age in a U.S. Representative Population-Based Study**

**Supplementary Material**

**Table of Contents**

[1. Supplementary Methods 3](#_Toc192670389)

[A. Additional Information on Variables 3](#_Toc192670390)

[i. Components of the CAIDE score: 3](#_Toc192670391)

[ii. Apolipoprotein E (APOE) ε4: 3](#_Toc192670392)

[iii. Blood Based Biomarkers: 3](#_Toc192670393)

[iv. Cognitive Function: 3](#_Toc192670394)

[v. Social origins score: 3](#_Toc192670395)

[vi. Recent inflammatory condition: 3](#_Toc192670396)

[vii. Race/Ethnicity: 4](#_Toc192670397)

[B. Sample Selection and Characteristics of Wave IV overall and Wave V biosample 5](#_Toc192670398)

[C. Supplementary Analysis 5](#_Toc192670399)

[i. Variation by life stage: 5](#_Toc192670400)

[ii. Inverse Probability of Sampling Weights for Wave V analyses: 5](#_Toc192670401)

[iii. Comparison of associations by APOE Genotype Coding: 5](#_Toc192670402)

[iv. Longitudinal analyses: 5](#_Toc192670403)

[D. Supplementary Tables and Figures 6](#_Toc192670404)

[Table S1. Proportion of Left-Censoring by Biomarker 6](#_Toc192670405)

[Figure S1. Wave IV Sample Selection 7](#_Toc192670406)

[Figure S2. Wave V Sample Selection 8](#_Toc192670407)

[Table S2. Weighted Sample Characteristics compared to Overall Study Population, National Longitudinal Study of Adolescent to Adult Health (Add Health) Waves IV-V 9](#_Toc192670408)

[Figure S3: Associations between CAIDE score/APOE Genotype and Cognitive Test Scores, Restricted to Participants with Data in Both Waves, National Longitudinal Study of Adolescent to Adult Health (Add Health) Wave IV and Wave V 11](#_Toc192670409)

[Figure S4. Association between CAIDE Score and Cognitive Test Scores, National Longitudinal Study of Adolescent to Adult Health (Add Health) Wave V (Inverse Probability of Sampling Weights Applied), N=529 12](#_Toc192670410)

[Figure S5. Association between APOE Genotype and Cognitive Test Scores, National Longitudinal Study of Adolescent to Adult Health (Add Health) Wave V (Inverse Probability of Sampling Weights Applied), N=1121 13](#_Toc192670411)

[Figure S6. Association between Amyloid, Tau, Neurodegeneration (ATN) Biomarkers and Cognitive Test Scores, 13](#_Toc192670412)

[Figure S7. Association between Immune Risk Biomarkers and Cognitive Test Scores, National Longitudinal Study of Adolescent to Adult Health (Add Health) Wave V (Inverse Probability of Sampling Weights Applied), N=588 14](#_Toc192670413)

[Figure S8. Association between ε4/ε4 Genotype and Cognitive Test Scores, National Longitudinal Study of Adolescent to Adult Health (Add Health) Wave IV and Wave V 15](#_Toc192670414)

[Figure S9. Association between Number of APOE ε4 alleles and Cognitive Test Scores, National Longitudinal Study of Adolescent to Adult Health (Add Health) Wave IV and Wave V 15](#_Toc192670415)

[Figure S10. Association between Wave IV Immune Risk/CAIDE Score and Wave V Cognitive Function Test Scores, Adjusting for Wave IV scores. Add Health Waves IV-V, N=378 16](#_Toc192670416)

[Figure S11. Association between Wave IV Immune Risk and CAIDE and Wave V Cognitive Function Test Scores. Add Health Waves IV-V, N=378 17](#_Toc192670417)

[2. Associations Presented in Main Manuscript Figures 1-4 18](#_Toc192670418)

[Table S3. Associations Presented in Figures 1-4 18](#_Toc192670419)

[Table S4. Associations Presented in Figure S3. 20](#_Toc192670420)

[3. References 22](#_Toc192670421)

# Supplementary Methods

## Additional Information on Variables

### Components of the CAIDE score:

Age at the time of the survey were categorized into tertiles (in months plus years) within the entire wave. The highest tertile, middle, and lowest tertile received a weighted score of 4, 3, and 0, respectively. Educational attainment is based on survey responses at each wave and categorized as high (college degree or higher, score=0), medium (some college and/or technical training, score=2), and low (high school diploma/GED or lower, score=3). Males received a score of 1, while females received a 0. SBP measurements were taken by trained personnel during the in-person exam. Following current guidelines, we averaged the second and third SBP measurements. Anyone with an SBP greater than 140 received a score of 2. During the in-person exam, field staff measured height in cm from shoeless participants standing on uncarpeted floors and recorded weight to the nearest 0.1 kg. BMI was computed as kg/m2, and obesity is indicated by a BMI above 30. Those categorized as obese receive a score of 2, while those who are not receive a score of 0. Total cholesterol is categorized into deciles within each wave. Those with a total cholesterol measurement in the highest decile were given a score of 2, while those in the bottom 9 deciles were given a 0. Lastly, the physical activity score is derived from a series of survey questions about the frequency of participation in certain activities in the past week. Responses could range from 0 to 7 or more times (0-7). We added the number of times the respondent reported over all these questions. They were considered active if they participated in 2 or more active activities in the past week. The choice of 2x per week as a threshold was based on the attempt to harmonize with the original creation of the CAIDE score. Finally, APOE status was defined by having at least one ε4 allele (i.e., those with APOE ε2/ε4, ε3/ε4, or ε4/ε4 phenotypes).

### Apolipoprotein E (APOE) ε4:

Saliva DNA collection occurred during Wave IV (96% consent rate). Archived samples (N=12,200) were eligible for genome-wide genotyping, with data available for N=9,974 participants after quality control procedures. Genomic data was further supplemented using DNA from venous blood collected during the Wave V biovisit for N=11,550 participants.

### **Blood Based Biomarkers:**

Values were log-transformed due to the skewness of biomarker concentration distributions. The values were then standardized to compare estimates on the same scale and account for different modes of sample collection between waves. A value of the limit of detection (LOD)/√2 was assigned to left-censored observations below the LOD. Of note, there was a high proportion of left-censoring for IL-10 in Wave IV (11.7%) and IL-1β in Wave V (29.8%). The development and validity of the assays used to assess inflammatory cytokines in dried blood spots have been described elsewhere^1^. In some cases, instrument software provides extrapolated values below the LLOD if it can be differentiated from 0. Table S1 shows the proportion of data that was below the LLOD’s and the proportion of data that was missing due to being below the LLOD (i.e. left-censored).

### **Cognitive Function**:

Immediate word recall involved participants being read a list of 15 words and repeating back as many as possible within 90 seconds. Delayed word recall required participants to recall as many words as possible from the same list of words after a few minutes, with 60 seconds to respond. Backward digit span consisted of FE’s reading a list of numbers and participants were asked to recite the span of numbers backwards, with the span length gradually increasing until an error occurred or a maximum of 7 digits. Participants were given two chances for each span length. All cognitive function scores were standardized into a z-score for analysis, with a higher score indicating higher memory cognition.

### **Social origins score**:

A factor score based on Wave I parental reports of education, occupation, household income, and household receipt of public assistance and was standardized to a z-score for analysis.

### **Recent inflammatory condition**:

This indicator measures included respondents who self-reported gum disease, active infection, injury, acute illness, and/or surgery in the past 4 weeks, and/or fever in the past 2 weeks. In Wave V, those who reported taking the following medications in the past four weeks were also included: cox-2 inhibitors, corticotrophines, glucocorticords, anti-rheumatics, anti-psoriatics, immunosuppressive agents or monoclonal antibodies. There was less specific information on medication in Wave IV so only those self-reporting inflammatory conditions were included.

### **Race/Ethnicity**:

We included the social construct of race and ethnicity as a covariate because there are documented racial and ethnic differentials in health, including conditions and biomarkers related to inflammation^2,3^, immunity^4^, cardiovascular health^5,6^, and disparities in Alzheimer’s Disease^7,8^. Therefore, disadvantaged minoritized groups are more likely to experience exposure to adverse environments and health care access inequities that are linked to both our biomarker exposures and cognitive function outcomes^9,10^. A race/ethnicity variable was constructed from the Wave V survey. Participants self-selected one or more boxes from a list including: “American Indian or Alaska Native”, “Asian”, “Black, African American”, “Hispanic”, “Pacific Islander”, “White”, and “Some other race or origin”. Those who chose one response were assigned to the corresponding category. Those who selected more than one option also answered a question on which category they most identified with and were assigned to that category. Anyone who skipped the question was assigned to the race/ethnicity they were categorized as in Wave I. Because some of the cell sizes for race and ethnicity were smaller than 10 in Wave V, some cell sizes were collapsed with others for masking and analyses.

## Sample Selection and Characteristics of Wave IV overall and Wave V biosample

Sample selection is shown in flowcharts given in Figures S1 and S2. Given these data span two waves and different combinations of subsamples, the number of participants included in each analysis varies. We assume those with inflammatory cytokine data (N=5,019) in Wave IV consist of a random subsample of Wave IV overall (N=14,800 with valid survey weights). We also assume the major selection mechanism for inclusion in the Wave V CAIDE and Blood-Based Biomarker samples was participation in the Wave V biovisit (referred to as the Wave V Biosample, N=5,269 with valid survey weights). Finally, we assume the Wave V Genetic sample is a subpopulation of Wave V overall (N=12,057 with valid survey weights). Descriptive statistics and analyses were weighted to account for sampling procedures, attrition and subpopulation analysis to make results generalizable to the U.S population of adults who were enrolled in middle school or high school in the 1994-1995 school year. To examine this assumption further, descriptive statistics of the overall Wave IV sample, the Wave V biosample, and the overall Wave V Sample are included below in Table S2 for comparison to the analytic samples. In relation to Wave IV overall, each weighted sample was similar on demographics and other relevant variables. The only exception was a slightly lower proportion of participants with a college degree or higher in the Blood-Based Biomarker sample (26% vs. 30%).  Compared to the Wave V biosample, the analytic samples had a lower proportion of females, a higher proportion of Black/African American participants, a lower proportion of those with a college degree or higher, a higher proportion of those with a recent inflammatory condition (17% vs. 14%), and higher median CAIDE scores. The samples were similar in other characteristics.

## Supplementary Analysis

### **Variation by life stage**:

To investigate how the strength of association between the CAIDE scores and cognitive function might vary at different life stages, we conducted the same cross-sectional linear regressions but restricted to the participants who were included in both waves (n=412 for CAIDE v1, n=406 for CAIDE v2 with APOE status included). Similarly, to assess how the association between APOE status and cognitive function might vary at different life stages, we completed linear regressions to assess the association between APOE status and Wave IV cognitive function and Wave V cognitive function among those with data in both waves (n=1063). Results are shown below in Figure S3.

### **Inverse Probability of Sampling Weights for Wave V analyses**:

Given there were some differences between the Wave V CAIDE and Blood-Based Biomarker Samples vs. the Wave V Biosample and the Wave V Genetic sample vs. the Wave V overall sample. we created inverse of sampling (IPS) weights based on the probability of being sampled into each Wave V sample given sex assigned at birth, education, race/ethnicity, and an indicator for inflammatory condition for each Wave V sample. Those IPS weights were combined with the appropriate survey weight and used in the same Wave V analyses. Additionally, we also added high systolic blood pressure, high BMI, and high total cholesterol to the model for the probability of being sampled into the Wave V CAIDE sample. The results are available below in Supplementary Figures S4-S7. Results appear quite similar and the addition of the weights does not change which associations were significant at an alpha level of 0.05. Results are shown in Figures S4-S7.

### Comparison of associations by APOE Genotype Coding:

We performed sensitivity analyses to assess the association between those who had 1) ε4/ε4 genotype vs. all others and 2) two ε4 alleles vs. one ε4 alleles vs. no ε4 alleles. We treated number of ε4 alleles as an ordinal variable. None of the associations were significant. Results are shown in Figures S8-S9.

### Longitudinal analyses:

We conducted two additional analyses to assess these relationships over time: 1) the associations with Wave IV risk factors with Wave V, adjusting for Wave IV cognitive function and 2) the associations between Wave IV risk factors and Wave V cognitive function, without adjusting for Wave IV cognitive function. None of the associations were not (note the small sample size). Results are shown in Figures S10-S11.

## Supplementary Tables and Figures

#### Table S1. Proportion of Left-Censoring by Biomarker

|  | **Biomarker** | **LLOD** | **N, % below LOD and extrapolated** | | **N, % missing due to left-censoring** | |
| --- | --- | --- | --- | --- | --- | --- |
| Wave IV (dried blood spots) | IL-6 | 0.4 | 356 | 9.0% | 130, | 3.0% |
|  | IL-8 | 0.2 | 0 | 0% | 10, | 0.3% |
|  | IL-10 | 0.4 | 358 | 10.3% | 476, | 11.7% |
|  | TNF-α | 0.9 | 5 | 0.1% | 24, | 0.6% |
| Wave V (venous blood) | IL-6 | 0.05 | 0 | 0% | 0 | 0% |
|  | IL-8 | 0.06 | 0 | 0% | 0 | 0% |
|  | IL-10 | 0.04 | 1 | 0.01% | 0 | 0% |
|  | TNF-α | 0.04 | 0 | 0% | 0 | 0% |
|  | IL-1β | 0.01 | 28 | 4.5% | 162 | 29.8% |
|  | NfL | 0.416 | 0 | 0% | 0 | 0% |
|  | Total Tau | 0.38 | 6 | 1.1% | 0 | 0% |

IL=Interleukin, TNF-α=Tumor Necrosis Factor alpha, NfL=Neurofilament Light

#### Figure S1. Wave IV Sample Selection

**
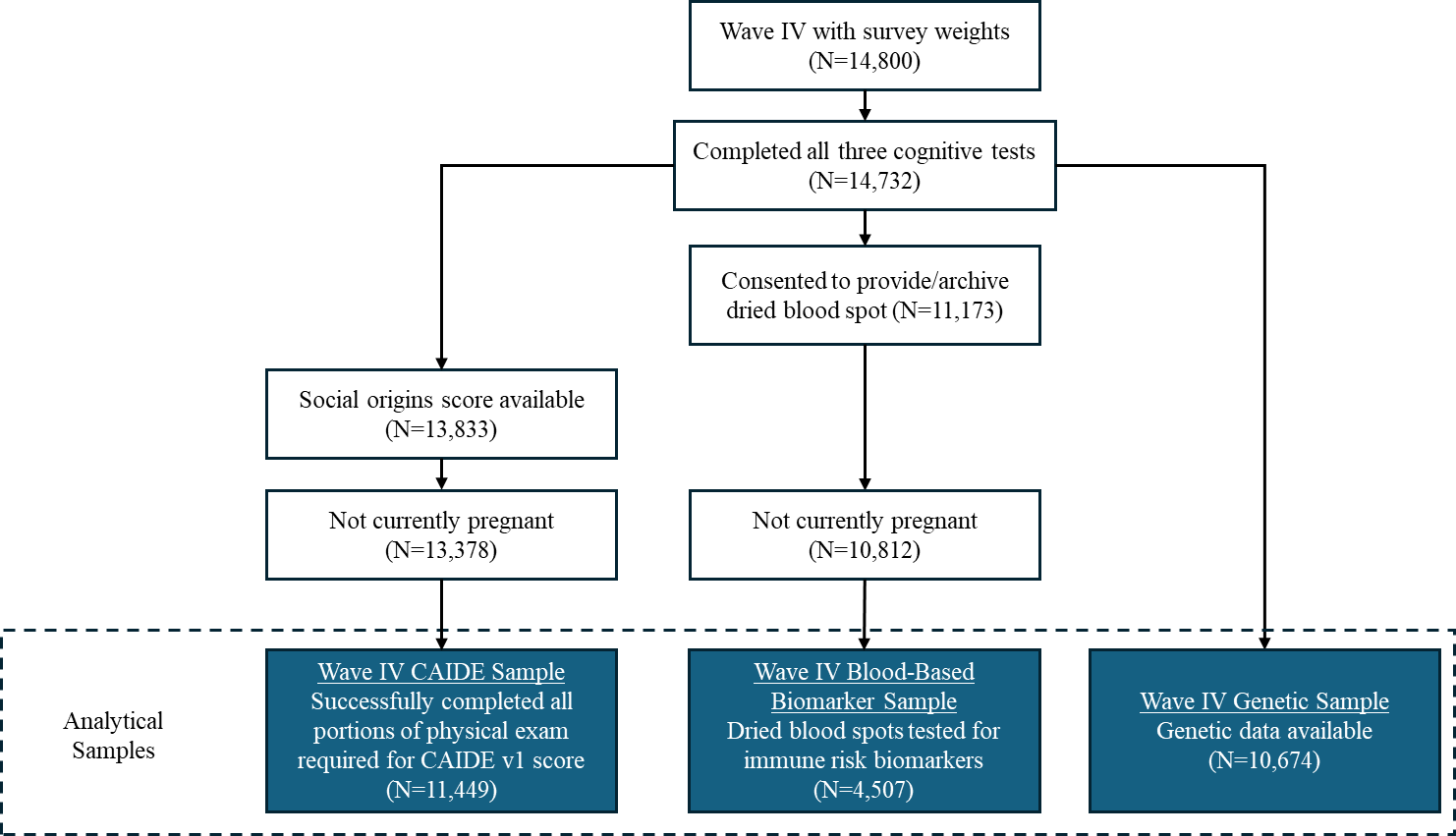
**

#### Figure S2. Wave V Sample Selection

**
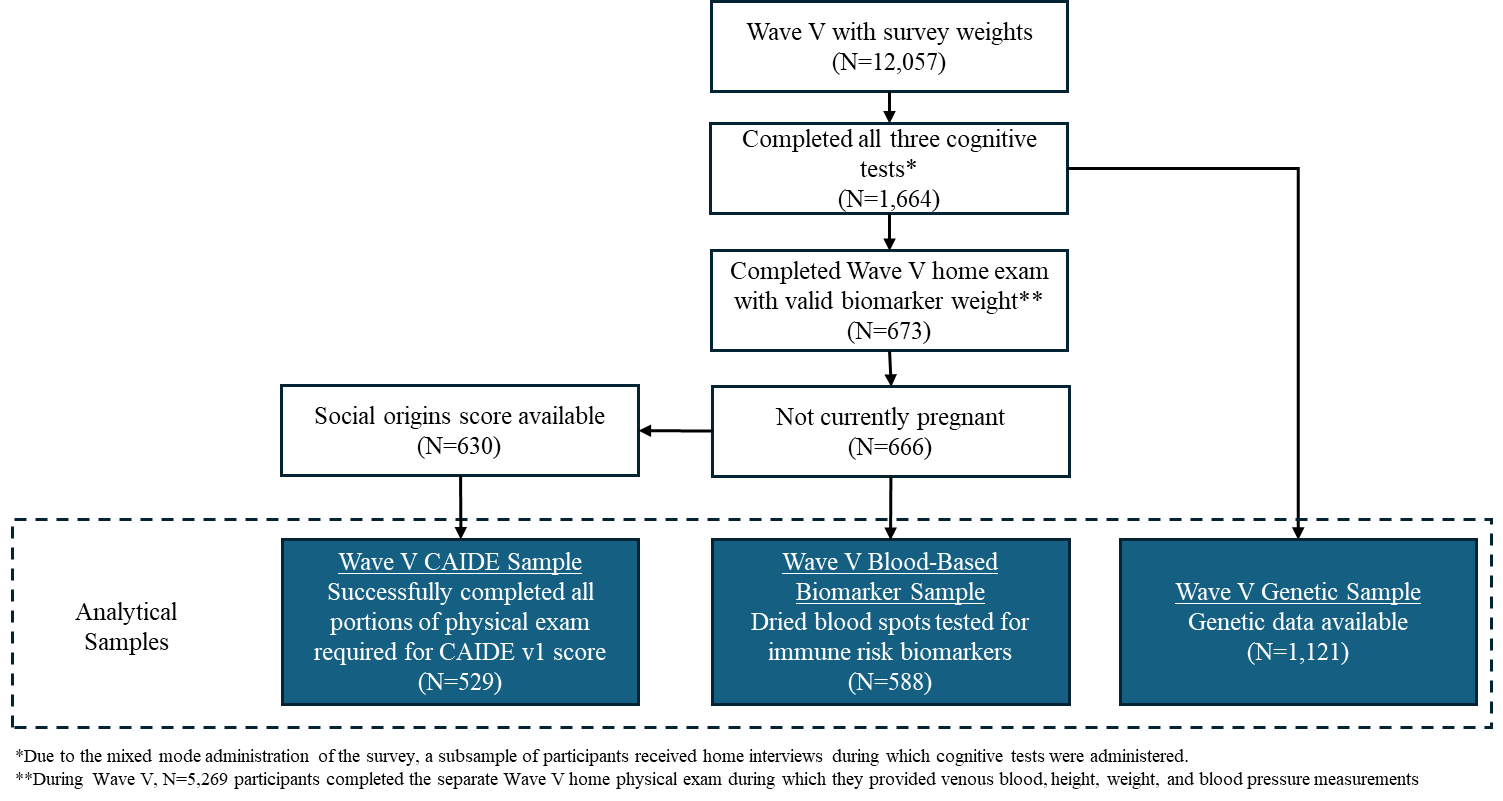
**

#### Table S2. Weighted Sample Characteristics compared to Overall Study Population, National Longitudinal Study of Adolescent to Adult Health (Add Health) Waves IV-V

|  |  | **Wave IV Overall Sample** | | **Wave V Biosample** | | **Wave V Overall Sample** | |
| --- | --- | --- | --- | --- | --- | --- | --- |
|  |  | *n=14,800* | | *n=5,269* | | *n=12,057* | |
| Age (years) | | 27.8 | (26.3, 29.3) | 37.4 | (35.9, 38.9) | 37.2 | (35.6, 38.7) |
| Sex | |  |  |  |  |  |  |
|  | Female | 7870, | 49.32% | 3171, | 50.5% | 5238, | 50.3% |
| Race | |  |  |  |  |  |  |
|  | American Indian or Alaska Native | 156, | 1.0% | 37, | 0.9% | 104, | 0.9% |
|  | Asian | 850, | 3.0% | 262, | 2.6% | 688, | 3.3% |
|  | Black, African American | 3197, | 15.9% | 1035, | 17.3% | 2410, | 15.9% |
|  | Hispanic | 2094, | 10.3% | 528, | 8.4% | 1485, | 9.4% |
|  | Pacific Islander | 77, | 0.3% | 27, | 0.2% | 90, | 0.4% |
|  | Some other race or origin | 50, | 0.3% | 17, | 0.4% | 49, | 0.3% |
|  | White | 8376, | 69.2% | 3363, | 70.2% | 7231, | 69.7% |
| Education | |  |  |  |  |  |  |
|  | College Degree or Higher | 4737, | 29.9% | 2449, | 41.3% | 4928, | 36.6% |
|  | Some College and/or Technical Training | 6521, | 42.9% | 2023, | 40.7% | 4871, | 41.3% |
|  | High School/GED or lower | 3538, | 27.2% | 796, | 18.1% | 2255, | 22.0% |
| Recent Inflammatory Condition^a^ | | 2252, | 15.8% | 734, | 14.0% | 734 | 5.8% |
| Social Origins Score^b^ | | 0.1 | (-0.8, 1.0) | 0.3 | (-0.6, 1.1) | 0.2 | (-0.7, 1.0) |
| CAIDE score v1^c^ | | 5.3 | (3.2, 7.2) | 4.9 | (2.9, 6.9) | NA^d^ | |
| CAIDE score v2^c^ (with APOE status) | | 6.8 | (4.3, 9.1) | 6.2 | (3.8, 8.7) |  |  |
|  | *missing* | *5226* |  | *918* |  |  |  |
| Systolic blood pressure > 140 mm Hg | | 1665, | 12.2% | 552, | 12.3% |  |  |
|  | *missing* | *501* |  | *217* |  |  |  |
| BMI > 30 kg/m2 | | 5320, | 36.4% | 2337, | 46.0% |  |  |
|  | *missing* | *233* |  | *102* |  |  |  |
| Total Cholesterol ≥ 90th percentile | | 1315, | 10.4% | 455, | 9.9% |  |  |
|  | *missing* | *1691* |  | *579* |  |  |  |
| Physical activity < 2x per week | | 3354, | 22.1% | 954, | 18.6% |  |  |
|  | *missing* | *14* |  | *36* |  |  |  |
| Smoking status | |  |  |  |  |  |  |
|  | Never smoker | 7729, | 48.0% | 3079, | 52.8% | 7020, | 53.0% |
|  | Ever smoker | 1739, | 12.5% | 1034, | 22.4% | 2287, | 20.9% |
|  | Current Smoker | 5332, | 39.5% | 1153, | 24.8% | 2731, | 26.2% |
|  | missing | *0* |  | *3* |  | *19* |  |
| Blood-based Biomarkers (log-transformed) | | *(n=5,019)* | |  |  |  |  |
|  | hsCRP (mg/L) | 0.7 | (-0.2, 1.7) | 0.6 | (-0.2, 1.5) | NA^d^ | |
|  | TNF-a (pg/mL) | 1.1 | (0.9, 1.3) | 0.9 | (0.7, 1.1) |  |  |
|  | IL-6 (pg/mL) | -0.2 | (-0.6, 0.3) | -0.4 | (-0.9, 0.1) |  |  |
|  | IL-10 (pg/mL) | -0.9 | (-1.4, -0.4) | -1.4 | (-1.8, -1.0) |  |  |
|  | IL-8 (pg/mL) | 4.3 | (4.0, 4.6) | 2.6 | (2.3, 3.1) |  |  |
|  | IL-1B (pg/mL) | NA |  | -3.7 | (5.0, -2.8) |  |  |
|  | NfL (pg/mL) | NA |  | 1.8 | (1.6, 2.1) |  |  |
|  | Total Tau (pg/mL) | NA |  | 0.8 | (0.4, 1.1) |  |  |
| APOE ε4 status | |  |  |  |  |  |  |
|  | ε4 carrier (ε2/ε4, ε3/ε4, or ε4/ε4) | 2957, | 27.4% | 1351, | 26.8% | 2321, | 27.1% |
|  | *missing* | *4091* |  | *301* |  | *3626* |  |
| Cognitive Function Tests | |  |  |  |  | *(n=1701)* | |
|  | Immediate Word Recall | 6.0 | (4.7, 7.4) | 5.7 | (4.4, 7.0) | 5.5 | (4.2, 6.9) |
|  | Delayed Word Recall | 4.6 | (3.3, 5.9) | 4.1 | (2.7, 5.5) | 4.0 | (2.7, 5.3) |
|  | Backwards Digit Span | 3.4 | (2.4, 4.8) | 3.5 | (2.4, 4.9) | 3.4 | (2.4, 4.7) |

Data are shown as N, survey-weighted % for categorical variables and weighted median, (IQR) for continuous variables.

CAIDE=Cardiovascular Risk Factors, Aging, and Incidence of Dementia, BMI=Body Mass Index, hsCRP=high sensitivity C-Reactive Protein, TNF-α=Tumor Necrosis Factor alpha, IL=Interleukin, NfL=Neurofilament Light, APOE=Apolipoprotein E

^a^Indicator of any of the following conditions: gum disease, active infection, injury, acute illness, and/or surgery in the past 4 weeks, and/or fever in the past 2 weeks. Reported use of cox-2 inhibitors, corticotrophines, glucocorticords, anti-rheumatics, anti-psoriatics, immunosuppressive agents or monoclonal antibodies in the past 4 weeks included in Wave V

^b^Factor score based on Wave I parental reports of education, occupation, household income, and household receipt of public assistance (standardized to a z-score for analysis).

^c^CAIDE score version 1 is a weighted sum of education, sex, age, total cholesterol, systolic blood pressure, body mass index, and physical activity. Version 2 includes the same variables and adds APOE ε4 carrier status.

^d^Factors requiring the separate Wave V biovisit are only shown for the Wave V Biosample

#### Figure S3: Associations between CAIDE score/APOE Genotype and Cognitive Test Scores, Restricted to Participants with Data in Both Waves, **National Longitudinal Study of Adolescent to Adult Health (Add Health)** Wave IV and Wave V

**
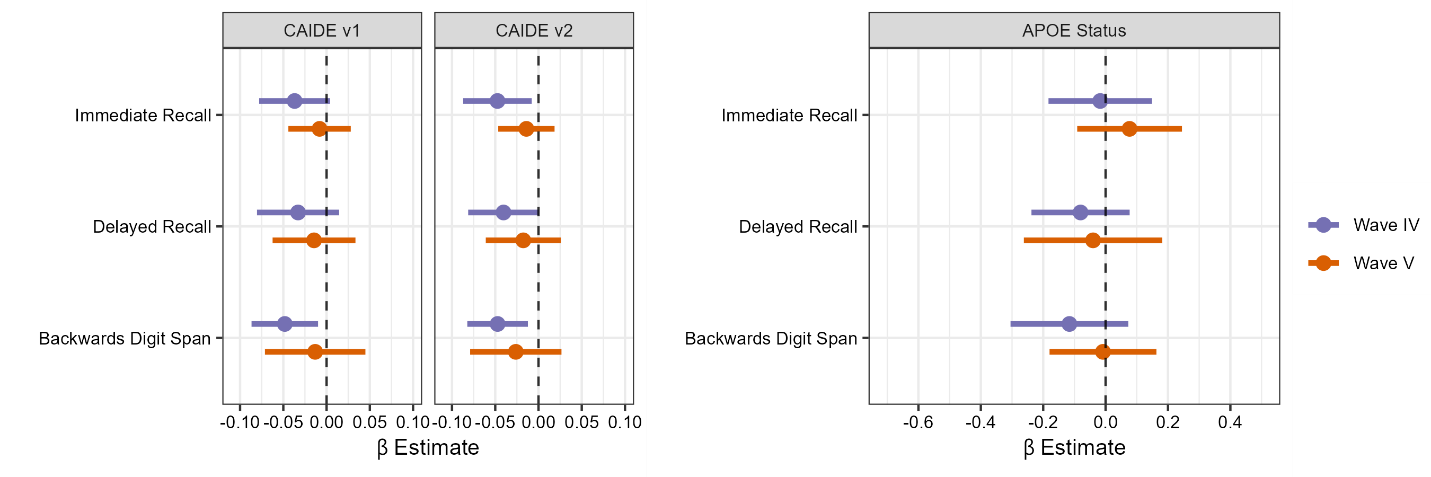
**

Wave IV was conducted in 2008 when participants had a median age of 28 years (IQR: 26-29 years), Wave V was conducted in 2016-2018 when participants had a median age of 38 (IQR: 36-39 years).

CAIDE=Cardiovascular Risk Factors, Aging, and Incidence of Dementia, APOE=Apolipoprotein E

APOE status defined as having at least one ε4 allele (i.e. those with APOE ε2/ε4, ε3/ε4, or ε4/ε4 phenotypes vs. ε2/ε2, ε3/ε3, or ε2/ε3).

CAIDE score version 1 (v1) is a weighted sum of education, sex, age, total cholesterol, systolic blood pressure, body mass index, and physical activity. Version 2 (v2) includes the same variables and adds APOE status.

Each panel shows the β estimate and 95% CI for cross-sectional survey-weighted linear regressions where the CAIDE scores/APOE status (ε4 carrier vs. non-carrier) are the independent variable and cognitive function tests scores are the dependent variable.

CAIDE models adjusted for race/ethnicity, early life socioeconomic status, and an indicator for inflammatory conditions. APOE models are adjusted for age and sex assigned at birth.

N=412 for CAIDE version 1, N=406 for CAIDE version 2, N=1063 for APOE status.

#### **Figure S4. Association between CAIDE Score and Cognitive Test Scores, National Longitudinal Study of Adolescent to Adult Health (Add Health) Wave V (Inverse Probability of Sampling Weights Applied), N=529**

**
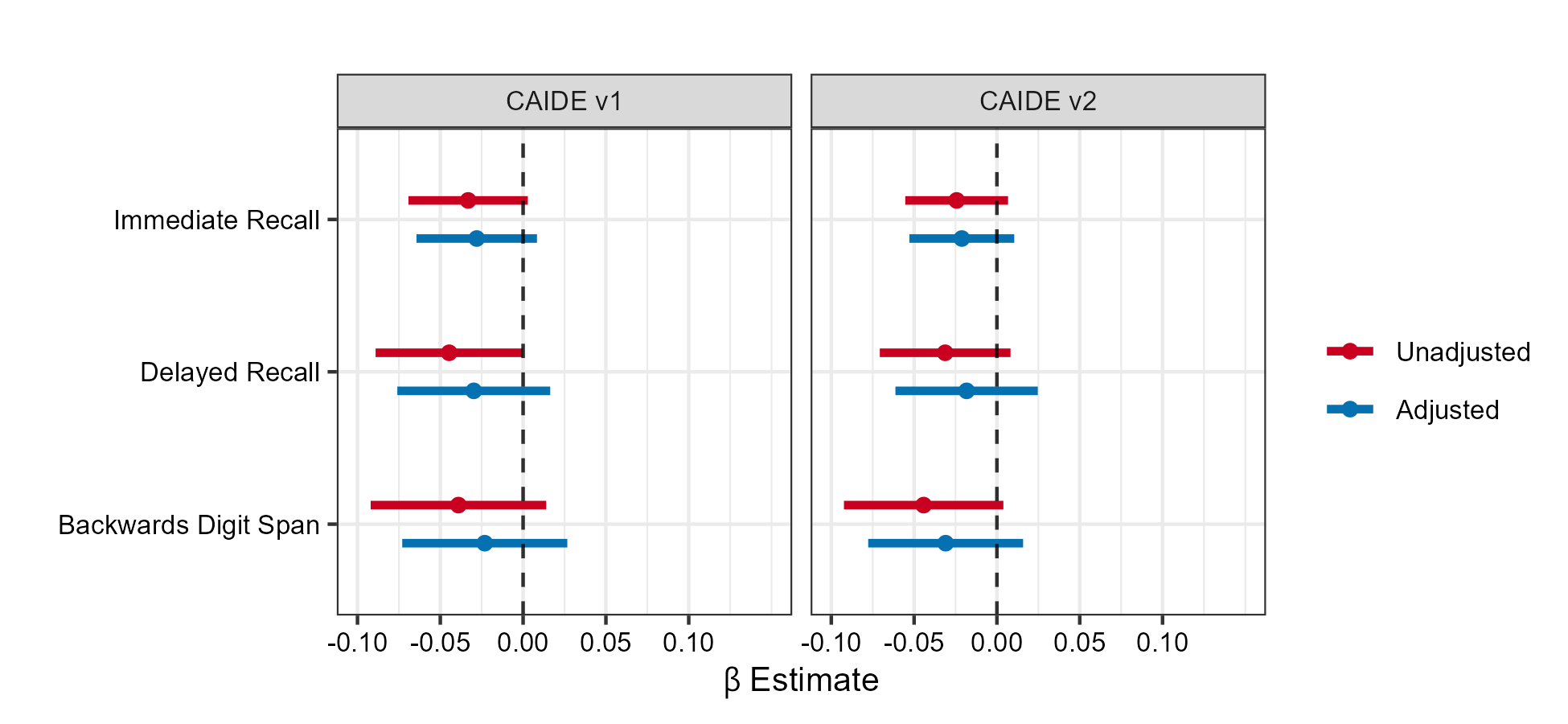
**

CAIDE=Cardiovascular Risk Factors, Aging, and Incidence of Dementia, APOE=Apolipoprotein E

CAIDE score version 1 (v1) is a weighted sum of education, sex, age, total cholesterol, systolic blood pressure, body mass index, and physical activity. Version 2 (v2) includes the same variables and adds APOE status.

APOE status defined as having at least one ε4 allele (i.e. those with APOE ε2/ε4, ε3/ε4, or ε4/ε4 phenotypes vs. ε2/ε2, ε3/ε3, or ε2/ε3).

Each panel shows the β estimates and 95% CIs for survey-weighted linear regressions where CAIDE scores are the independent variable and cognitive function tests scores are the dependent variable.

Models are adjusted for race/ethnicity, social origins score, an indicator for recent inflammatory conditions, and smoking status

Wave V: N=529 for CAIDE score v1, N=520 for the CAIDE score v2.

#### Figure S5. Association between APOE Genotype and Cognitive Test Scores, **National Longitudinal Study of Adolescent to Adult Health (Add Health)** Wave V (Inverse Probability of Sampling Weights Applied), N=1121


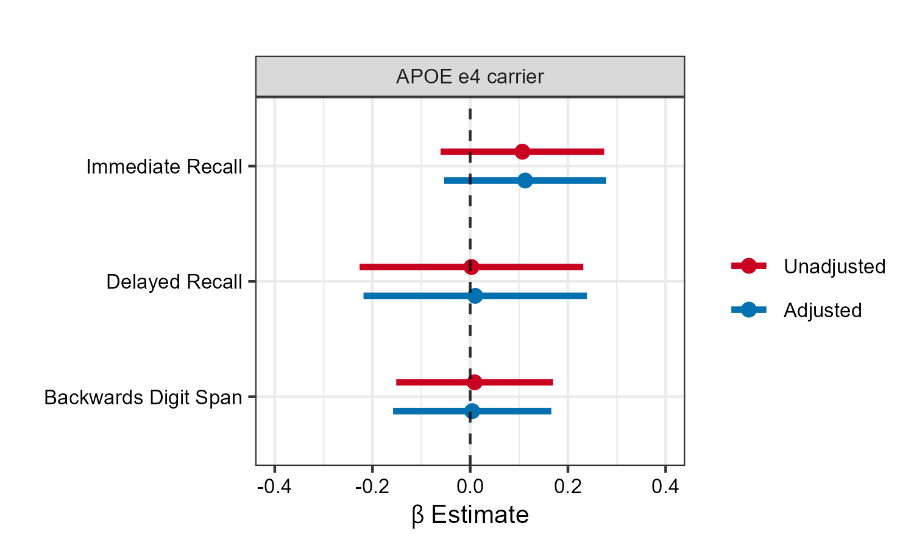


APOE=Apolipoprotein E

APOEe4 carrier status defined as having at least one ε4 allele (i.e. those with APOE ε2/ε4, ε3/ε4, or ε4/ε4 phenotypes vs. ε2/ε2, ε3/ε3, or ε2/ε3).

Each panel shows the β estimates and 95% CIs for survey-weighted linear regressions where APOE e4 carrier status (carrier vs. non-carrier) is the independent variable and cognitive function tests scores are the dependent variable.

Models adjusted sex assigned at birth and age.

Figure S6. Association between Amyloid, Tau, Neurodegeneration (ATN) Biomarkers and Cognitive Test Scores, National Longitudinal Study of Adolescent to Adult Health (Add Health) Wave V (Inverse Probability of Sampling Weights Applied), N=588**
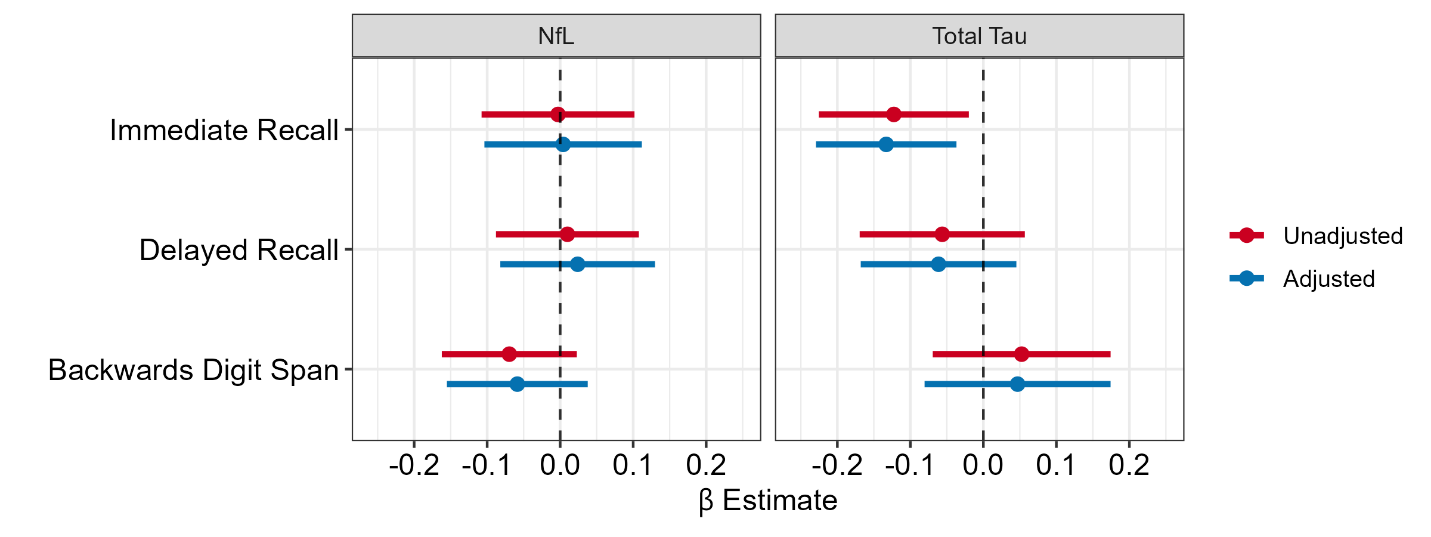
**

NfL=Neurofilament Light

Each panel shows the β estimate and 95% CI for survey-weighted linear regressions where Wave V blood-based Amyloid, Tau, Neurodegeneration (ATN) biomarker concentrations are the independent variable and Wave V cognitive function tests scores are the dependent variable.

Models adjusted for race/ethnicity, education, sex assigned at birth, age, an indicator for inflammatory conditions, and smoking status

NfL N=588, total Tau N=584

#### Figure S7. Association between Immune Risk Biomarkers and Cognitive Test Scores, **National Longitudinal Study of Adolescent to Adult Health (Add Health)** Wave V (Inverse Probability of Sampling Weights Applied), N=588

**
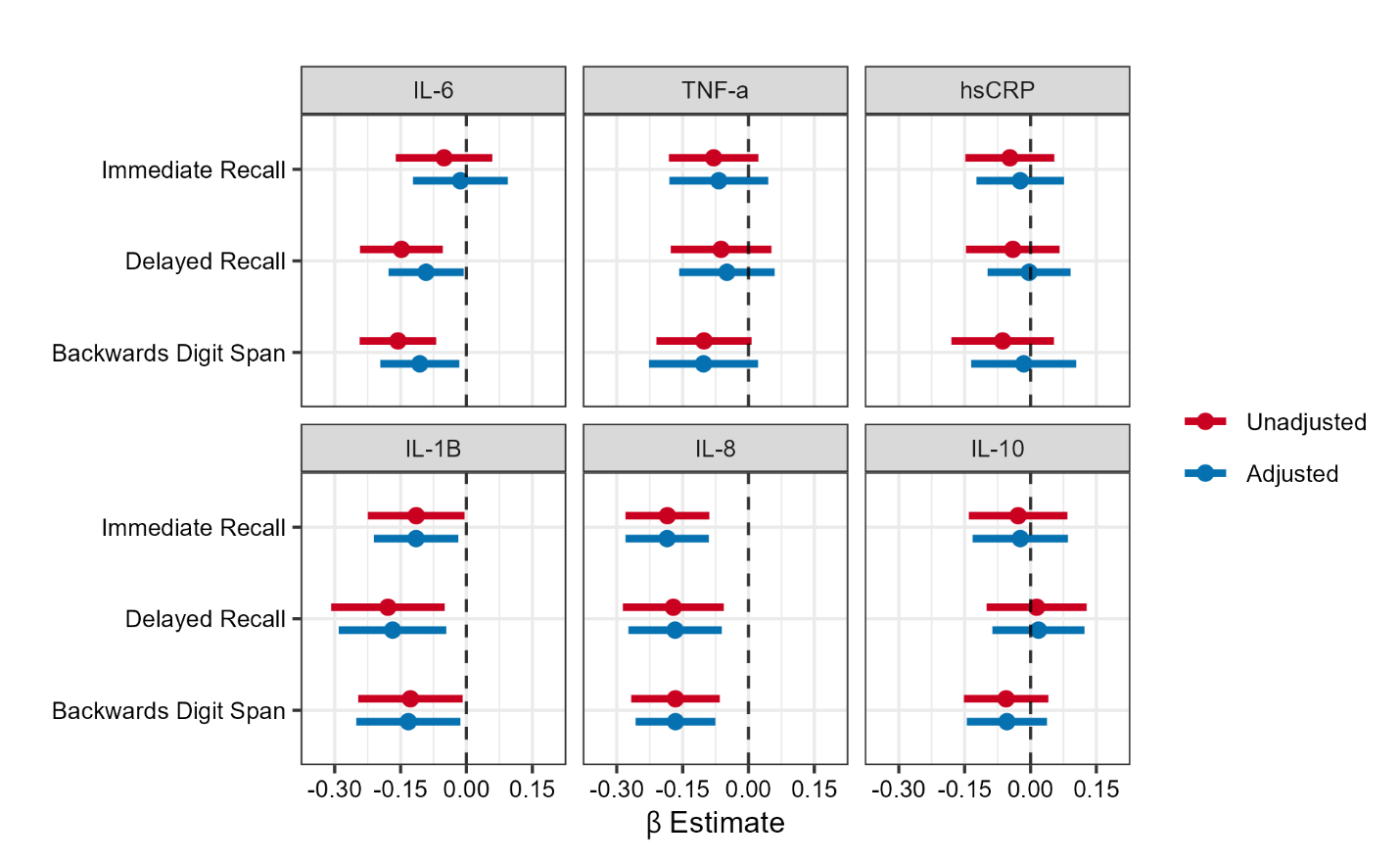
**

IL=Interleukin, TNF-α=Tumor Necrosis Factor alpha, hsCRP=high sensitivity C-Reactive Protein

Each panel shows the β estimates and 95% CIs for cross-sectional survey-weighted linear regressions where immune risk biomarker concentrations are the independent variable and cognitive function tests scores are the dependent variable.

Models adjusted for race/ethnicity, education, sex assigned at birth, age, an indicator for inflammatory conditions, and smoking status

Wave V (serum): N=567 for hsCRP, N=588 for all other biomarkers.

#### Figure S8. **Association between ε4/ε4 Genotype and Cognitive Test Scores, National Longitudinal Study of Adolescent to Adult Health (Add Health) Wave IV and Wave V**


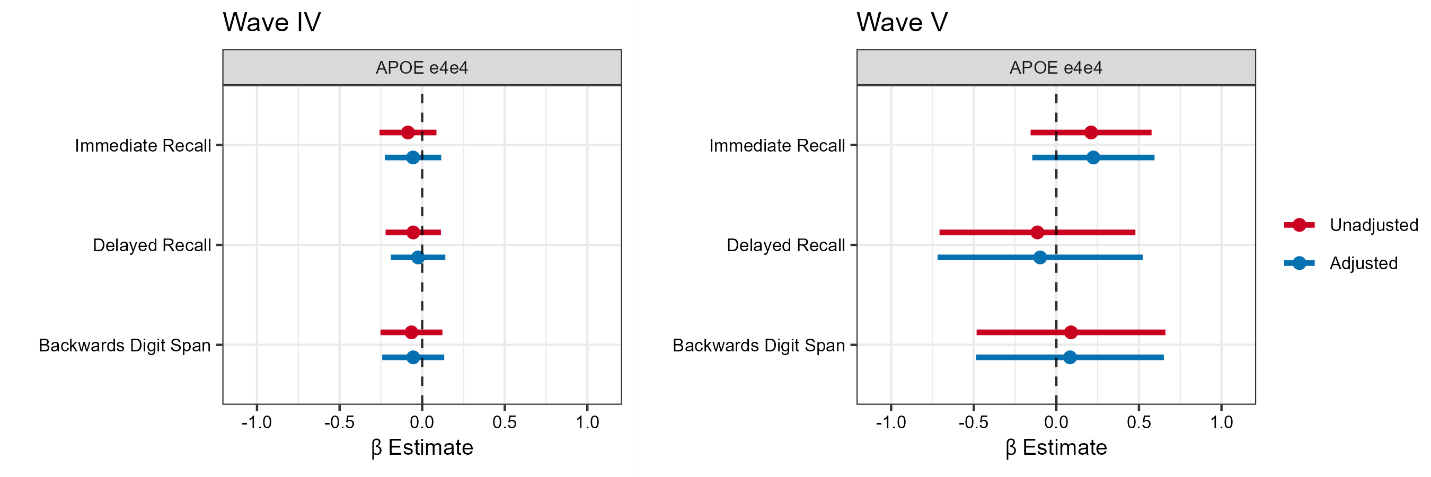


APOE e4e4=Apolipoprotein E e4/e4 genotype

Wave IV was conducted in 2008 when participants had a median age of 28 years (IQR: 26-29 years), Wave V was conducted in 2016-2018 when participants had a median age of 38 (IQR: 36-39 years).

Each panel shows the β estimates and 95% CIs for survey-weighted linear regressions comparing ε4/ε4 homozygotes vs. those with ≤1 ε4 alleles.

Models adjusted sex assigned at birth and age.

Wave IV N=10,674 (N=271 with ε4/ε4)

Wave V N=1,121 (N=31 with ε4/ε4)

#### Figure S9. **Association between Number of APOE ε4 alleles and Cognitive Test Scores, National Longitudinal Study of Adolescent to Adult Health (Add Health) Wave IV and Wave V**


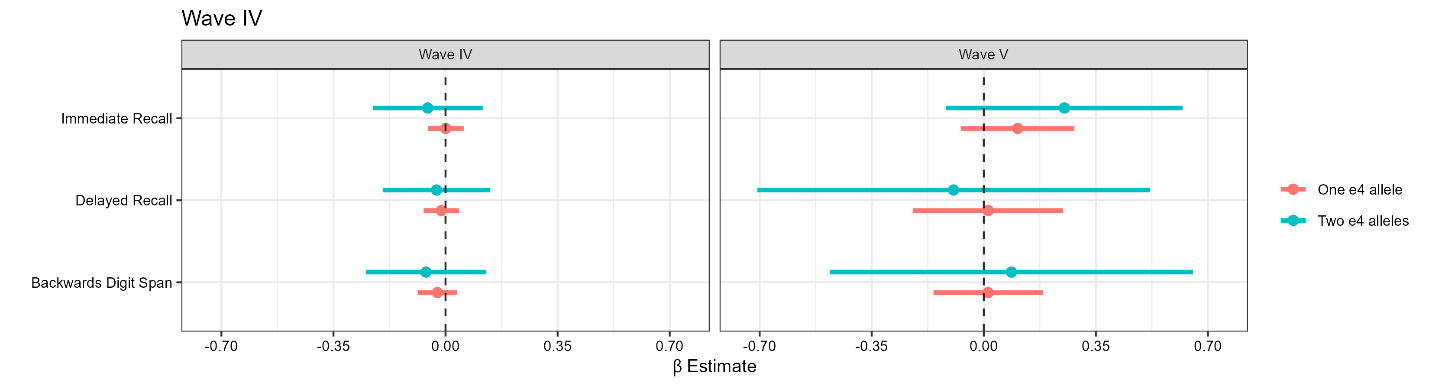


APOE=Apolipoprotein E

Wave IV was conducted in 2008 when participants had a median age of 28 years (IQR: 26-29 years), Wave V was conducted in 2016-2018 when participants had a median age of 38 (IQR: 36-39 years).

Each panel shows the β estimates and 95% CIs for survey-weighted adjusted linear regressions comparing one e4 alleles vs. non-carriers and two e4 alleles vs. non-carriers.

All models adjusted sex assigned at birth and age.

Wave IV N=10,674

Wave V N=1,121

Figure S10. Association between Wave IV Immune Risk/CAIDE Score and Wave V Cognitive Function Test Scores, Adjusting for Wave IV scores. Add Health Waves IV-V, N=378 **
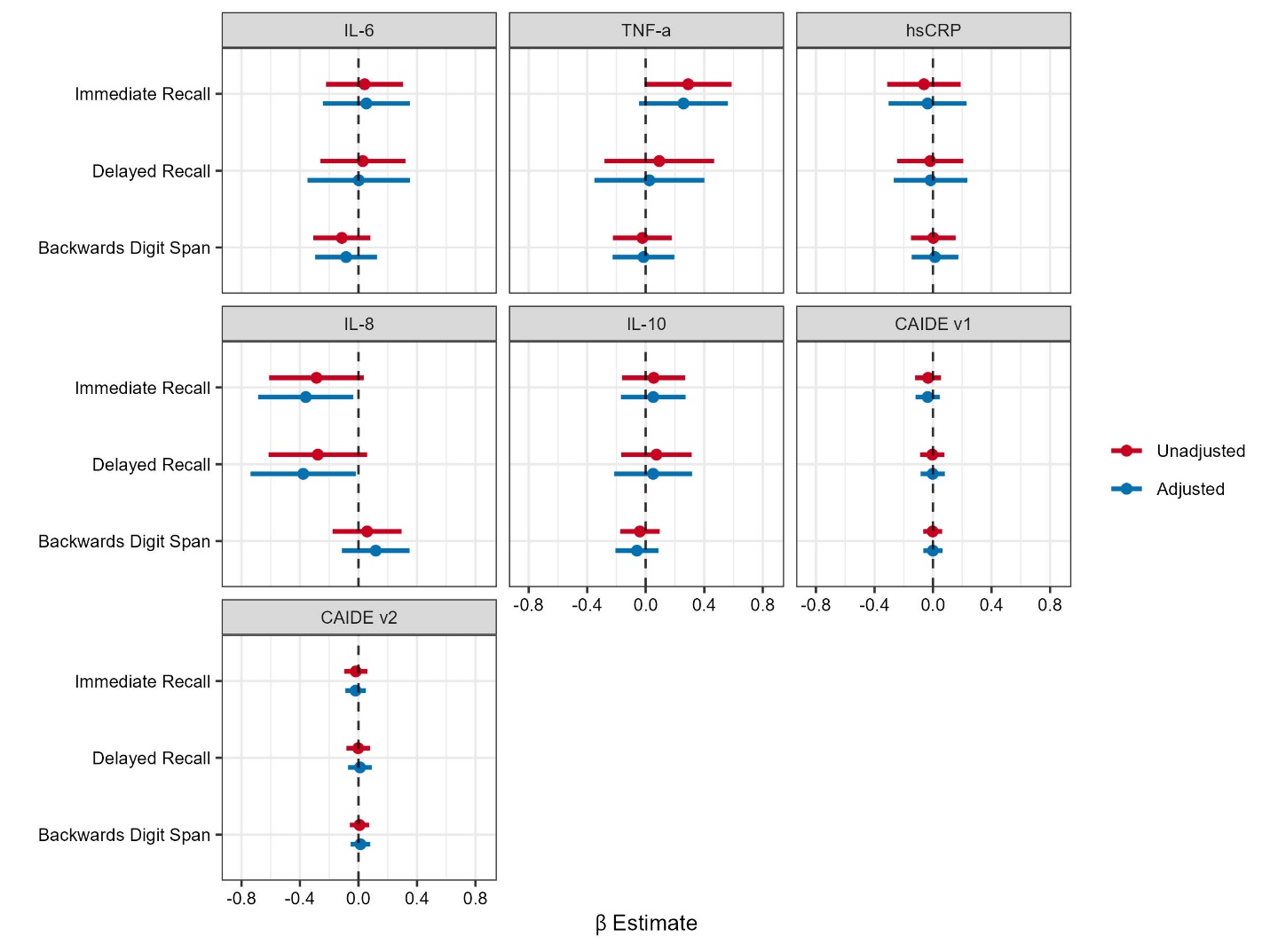
**

Wave IV was conducted in 2008 when participants had a median age of 28 years (IQR: 26-29 years), Wave V was conducted in 2016-2018 when participants had a median age of 38 (IQR: 36-39 years).

CAIDE=Cardiovascular Risk Factors, Aging, and Incidence of Dementia, APOE=Apolipoprotein E

CAIDE score version 1 (v1) is a weighted sum of education, sex, age, total cholesterol, systolic blood pressure, body mass index, and physical activity. Version 2 (v2) includes the same variables and adds APOE status. APOE status defined as having at least one ε4 allele (i.e. those with APOE ε2/ε4, ε3/ε4, or ε4/ε4 phenotypes vs. ε2/ε2, ε3/ε3, or ε2/ε3).

CAIDE v1 and Wave IV biomarker models N=378, hsCRP N=376

CAIDE v2 N=330

Immune risk biomarker models adjusted for race/ethnicity, education, sex assigned at birth, an indicator for inflammatory conditions, and smoking status

CAIDE models adjusted for race/ethnicity, social origins score, an indicator for inflammatory conditions, and smoking status

#### Figure S11. Association between Wave IV Immune Risk and CAIDE and Wave V Cognitive Function Test Scores. Add Health Waves IV-V, N=378


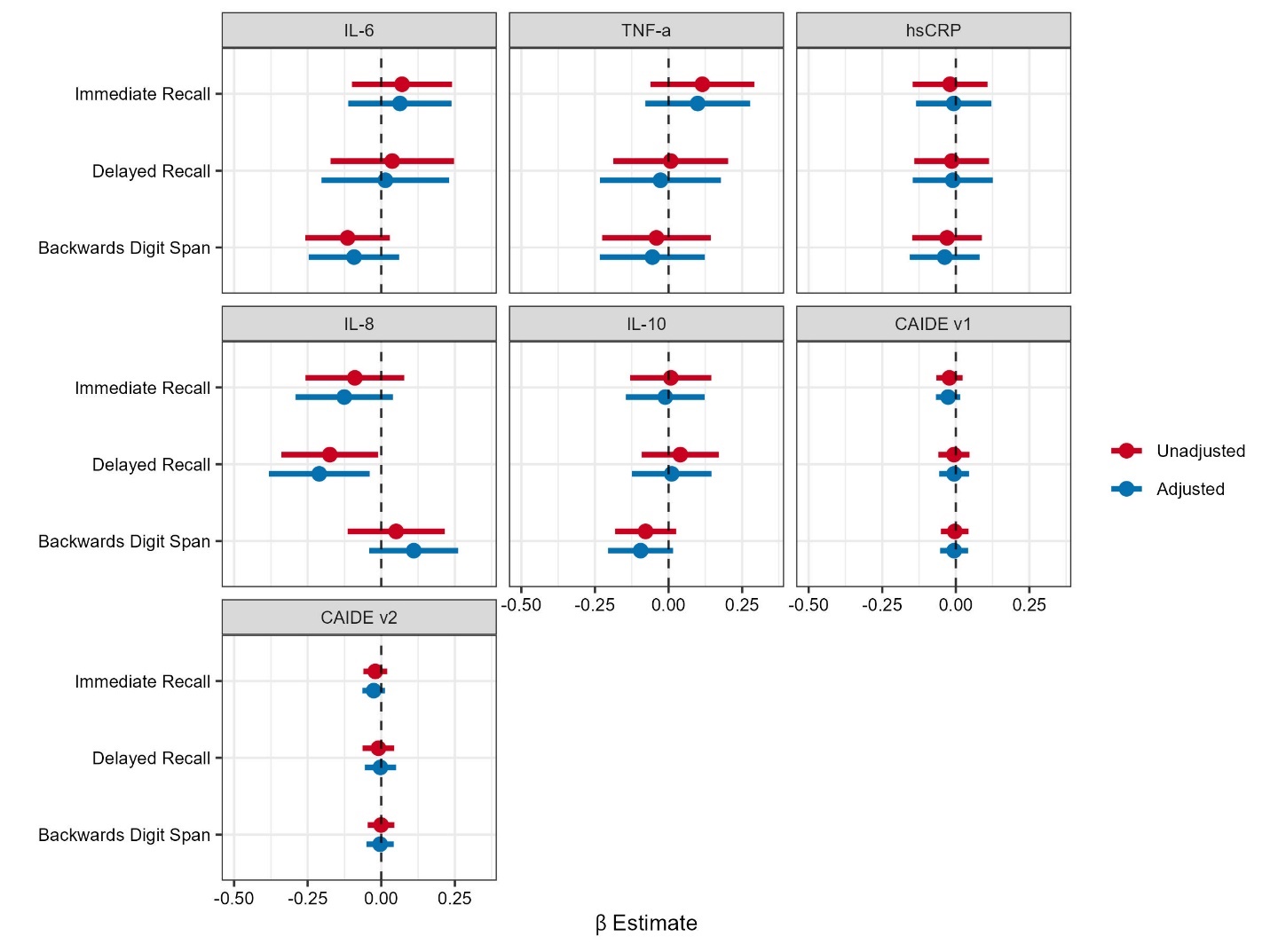


Wave IV was conducted in 2008 when participants had a median age of 28 years (IQR: 26-29 years), Wave V was conducted in 2016-2018 when participants had a median age of 38 (IQR: 36-39 years).

CAIDE=Cardiovascular Risk Factors, Aging, and Incidence of Dementia, APOE=Apolipoprotein E

CAIDE score version 1 (v1) is a weighted sum of education, sex, age, total cholesterol, systolic blood pressure, body mass index, and physical activity. Version 2 (v2) includes the same variables and adds APOE status. APOE status defined as having at least one ε4 allele (i.e. those with APOE ε2/ε4, ε3/ε4, or ε4/ε4 phenotypes vs. ε2/ε2, ε3/ε3, or ε2/ε3).

CAIDE v1 and Wave IV biomarker models N=380, hsCRP N=378

CAIDE v2 N=331

Immune risk biomarker models adjusted for race/ethnicity, education, sex assigned at birth, an indicator for inflammatory conditions, and smoking status

CAIDE models adjusted for race/ethnicity, social origins score, an indicator for inflammatory conditions, and smoking status

# Associations Presented in Main Manuscript Figures 1-4

β estimates and 95% confidence intervals for all analyses presented in the main manuscript in Figures 1-4 can be found in Table S3 below. Estimates and 95% confidence intervals for analyses presented in Supplementary Figure S3 are shown in Table S4. Estimates for all other associations presented in Supplementary Analyses (Supplementary Figures S4-S12 are available upon request.

#### Table S3. Associations Presented in Figures 1-4

| **Wave** | **Independent Variable** | **Cognitive Domain** | **N** | **Model** | **Beta Estimate** | **95% CI** | | |
| --- | --- | --- | --- | --- | --- | --- | --- | --- |
| **CAIDE Scores** | | | | | | | |  |
| IV | CAIDE v1 | Backwards Digit Span | 11449 | unadjusted | -0.05 | -0.06 | -0.04 | |
| IV | CAIDE v1 | Backwards Digit Span | 11449 | adjusted | -0.03 | -0.04 | -0.02 | |
| IV | CAIDE v1 | Delayed Recall | 11449 | unadjusted | -0.06 | -0.07 | -0.05 | |
| IV | CAIDE v1 | Delayed Recall | 11449 | adjusted | -0.05 | -0.06 | -0.03 | |
| IV | CAIDE v1 | Immediate Recall | 11449 | unadjusted | -0.06 | -0.07 | -0.05 | |
| IV | CAIDE v1 | Immediate Recall | 11449 | adjusted | -0.05 | -0.06 | -0.03 | |
| IV | CAIDE v2 | Backwards Digit Span | 8685 | unadjusted | -0.05 | -0.06 | -0.04 | |
| IV | CAIDE v2 | Backwards Digit Span | 8685 | adjusted | -0.03 | -0.04 | -0.02 | |
| IV | CAIDE v2 | Delayed Recall | 8685 | unadjusted | -0.05 | -0.06 | -0.04 | |
| IV | CAIDE v2 | Delayed Recall | 8685 | adjusted | -0.04 | -0.05 | -0.03 | |
| IV | CAIDE v2 | Immediate Recall | 8685 | unadjusted | -0.05 | -0.06 | -0.04 | |
| IV | CAIDE v2 | Immediate Recall | 8685 | adjusted | -0.04 | -0.05 | -0.03 | |
| V | CAIDE v1 | Backwards Digit Span | 529 | unadjusted | -0.04 | -0.09 | 0.01 | |
| V | CAIDE v1 | Backwards Digit Span | 529 | adjusted | -0.02 | -0.07 | 0.03 | |
| V | CAIDE v1 | Delayed Recall | 529 | unadjusted | -0.04 | -0.09 | 0.00 | |
| V | CAIDE v1 | Delayed Recall | 529 | adjusted | -0.03 | -0.08 | 0.02 | |
| V | CAIDE v1 | Immediate Recall | 529 | unadjusted | -0.03 | -0.07 | 0.00 | |
| V | CAIDE v1 | Immediate Recall | 529 | adjusted | -0.03 | -0.06 | 0.01 | |
| V | CAIDE v2 | Backwards Digit Span | 520 | unadjusted | -0.04 | -0.09 | 0.00 | |
| V | CAIDE v2 | Backwards Digit Span | 520 | adjusted | -0.03 | -0.08 | 0.02 | |
| V | CAIDE v2 | Delayed Recall | 520 | unadjusted | -0.03 | -0.07 | 0.01 | |
| V | CAIDE v2 | Delayed Recall | 520 | adjusted | -0.02 | -0.06 | 0.02 | |
| V | CAIDE v2 | Immediate Recall | 520 | unadjusted | -0.02 | -0.06 | 0.01 | |
| V | CAIDE v2 | Immediate Recall | 520 | adjusted | -0.02 | -0.05 | 0.01 | |
| **APOE status (e4 carrier vs. non-carrier)** | | | | | | | |  |
| IV | e4 carrier | Backwards Digit Span | 10674 | adjusted | -0.03 | -0.09 | 0.03 | |
| IV | e4 carrier | Backwards Digit Span | 10674 | unadjusted | -0.03 | -0.09 | 0.03 | |
| IV | e4 carrier | Delayed Recall | 10674 | adjusted | -0.02 | -0.07 | 0.04 | |
| IV | e4 carrier | Delayed Recall | 10674 | unadjusted | -0.01 | -0.07 | 0.04 | |
| IV | e4 carrier | Immediate Recall | 10674 | adjusted | -0.01 | -0.06 | 0.05 | |
| IV | e4 carrier | Immediate Recall | 10674 | unadjusted | 0.00 | -0.06 | 0.05 | |
| V | e4 carrier | Backwards Digit Span | 1121 | adjusted | 0.03 | -0.14 | 0.19 | |
| V | e4 carrier | Backwards Digit Span | 1121 | unadjusted | 0.02 | -0.14 | 0.19 | |
| V | e4 carrier | Delayed Recall | 1121 | adjusted | 0.00 | -0.22 | 0.21 | |
| V | e4 carrier | Delayed Recall | 1121 | unadjusted | 0.00 | -0.21 | 0.22 | |
| V | e4 carrier | Immediate Recall | 1121 | adjusted | 0.12 | -0.05 | 0.28 | |
| V | e4 carrier | Immediate Recall | 1121 | adjusted | 0.12 | -0.04 | 0.29 | |
| IV | e4e4 homozygote | Backwards Digit Span | 10674 | unadjusted | -0.06 | -0.25 | 0.12 | |
| IV | e4e4 homozygote | Backwards Digit Span | 10674 | adjusted | -0.05 | -0.24 | 0.13 | |
| IV | e4e4 homozygote | Delayed Recall | 10674 | unadjusted | -0.05 | -0.22 | 0.11 | |
| IV | e4e4 homozygote | Delayed Recall | 10674 | adjusted | -0.02 | -0.19 | 0.14 | |
| IV | e4e4 homozygote | Immediate Recall | 10674 | unadjusted | -0.09 | -0.26 | 0.09 | |
| IV | e4e4 homozygote | Immediate Recall | 10674 | adjusted | -0.06 | -0.23 | 0.12 | |
| V | e4e4 homozygote | Backwards Digit Span | 1121 | unadjusted | 0.09 | -0.48 | 0.66 | |
| V | e4e4 homozygote | Backwards Digit Span | 1121 | adjusted | 0.08 | -0.48 | 0.65 | |
| V | e4e4 homozygote | Delayed Recall | 1121 | unadjusted | -0.11 | -0.71 | 0.48 | |
| V | e4e4 homozygote | Delayed Recall | 1121 | adjusted | -0.10 | -0.72 | 0.52 | |
| V | e4e4 homozygote | Immediate Recall | 1121 | unadjusted | 0.21 | -0.16 | 0.58 | |
| V | e4e4 homozygote | Immediate Recall | 1121 | adjusted | 0.22 | -0.15 | 0.59 | |
| **ATN Biomarkers** | | | | | | | |  |
| V | NfL | Backwards Digit Span | 588 | unadjusted | -0.07 | -0.16 | 0.02 | |
| V | NfL | Backwards Digit Span | 588 | adjusted | -0.06 | -0.16 | 0.04 | |
| V | NfL | Delayed Recall | 588 | unadjusted | 0.01 | -0.09 | 0.11 | |
| V | NfL | Delayed Recall | 588 | adjusted | 0.02 | -0.08 | 0.13 | |
| V | NfL | Immediate Recall | 588 | unadjusted | 0 | -0.11 | 0.10 | |
| V | NfL | Immediate Recall | 588 | adjusted | 0 | -0.10 | 0.11 | |
| V | total Tau | Backwards Digit Span | 584 | unadjusted | 0.05 | -0.07 | 0.17 | |
| V | total Tau | Backwards Digit Span | 584 | adjusted | 0.05 | -0.08 | 0.17 | |
| V | total Tau | Delayed Recall | 584 | unadjusted | -0.06 | -0.17 | 0.06 | |
| V | total Tau | Delayed Recall | 584 | adjusted | -0.06 | -0.17 | 0.05 | |
| V | total Tau | Immediate Recall | 584 | unadjusted | -0.12 | -0.23 | -0.02 | |
| V | total Tau | Immediate Recall | 584 | adjusted | -0.13 | -0.23 | -0.04 | |
| **Immune Biomarkers** | | | | | | | |  |
| IV | IL-6 | Backwards Digit Span | 4507 | unadjusted | -0.08 | -0.13 | -0.04 | |
| IV | IL-6 | Backwards Digit Span | 4507 | adjusted | -0.04 | -0.08 | 0.00 | |
| IV | IL-6 | Delayed Recall | 4507 | unadjusted | -0.02 | -0.06 | 0.03 | |
| IV | IL-6 | Delayed Recall | 4507 | adjusted | 0.00 | -0.04 | 0.05 | |
| IV | IL-6 | Immediate Recall | 4507 | unadjusted | -0.02 | -0.07 | 0.02 | |
| IV | IL-6 | Immediate Recall | 4507 | adjusted | -0.01 | -0.05 | 0.04 | |
| IV | IL-10 | Backwards Digit Span | 4507 | unadjusted | 0.00 | -0.05 | 0.04 | |
| IV | IL-10 | Backwards Digit Span | 4507 | adjusted | -0.01 | -0.06 | 0.03 | |
| IV | IL-10 | Delayed Recall | 4507 | unadjusted | -0.03 | -0.08 | 0.01 | |
| IV | IL-10 | Delayed Recall | 4507 | adjusted | -0.05 | -0.09 | -0.01 | |
| IV | IL-10 | Immediate Recall | 4507 | unadjusted | 0.00 | -0.03 | 0.04 | |
| IV | IL-10 | Immediate Recall | 4507 | adjusted | -0.01 | -0.04 | 0.02 | |
| IV | IL-8 | Backwards Digit Span | 4507 | unadjusted | -0.05 | -0.11 | 0.00 | |
| IV | IL-8 | Backwards Digit Span | 4507 | adjusted | -0.02 | -0.07 | 0.03 | |
| IV | IL-8 | Delayed Recall | 4507 | unadjusted | -0.04 | -0.09 | 0.01 | |
| IV | IL-8 | Delayed Recall | 4507 | adjusted | -0.01 | -0.06 | 0.04 | |
| IV | IL-8 | Immediate Recall | 4507 | unadjusted | -0.04 | -0.09 | 0.00 | |
| IV | IL-8 | Immediate Recall | 4507 | adjusted | -0.01 | -0.06 | 0.03 | |
| IV | TNF-α | Backwards Digit Span | 4507 | unadjusted | -0.04 | -0.08 | 0.00 | |
| IV | TNF-α | Backwards Digit Span | 4507 | adjusted | -0.03 | -0.07 | 0.01 | |
| IV | TNF-α | Delayed Recall | 4507 | unadjusted | -0.02 | -0.06 | 0.02 | |
| IV | TNF-α | Delayed Recall | 4507 | adjusted | -0.02 | -0.06 | 0.01 | |
| IV | TNF-α | Immediate Recall | 4507 | unadjusted | -0.01 | -0.05 | 0.03 | |
| IV | TNF-α | Immediate Recall | 4507 | adjusted | -0.01 | -0.04 | 0.03 | |
| IV | hsCRP | Backwards Digit Span | 4485 | unadjusted | -0.08 | -0.12 | -0.04 | |
| IV | hsCRP | Backwards Digit Span | 4485 | adjusted | -0.04 | -0.08 | 0.00 | |
| IV | hsCRP | Delayed Recall | 4485 | unadjusted | 0.02 | -0.02 | 0.06 | |
| IV | hsCRP | Delayed Recall | 4485 | adjusted | 0.02 | -0.01 | 0.06 | |
| IV | hsCRP | Immediate Recall | 4485 | unadjusted | 0.00 | -0.04 | 0.03 | |
| IV | hsCRP | Immediate Recall | 4485 | adjusted | 0.00 | -0.03 | 0.04 | |
| V | hsCRP | Backwards Digit Span | 567 | unadjusted | -0.06 | -0.18 | 0.05 | |
| V | hsCRP | Backwards Digit Span | 567 | adjusted | -0.02 | -0.14 | 0.10 | |
| V | hsCRP | Delayed Recall | 567 | unadjusted | -0.04 | -0.15 | 0.07 | |
| V | hsCRP | Delayed Recall | 567 | adjusted | 0 | -0.10 | 0.09 | |
| V | hsCRP | Immediate Recall | 567 | unadjusted | -0.05 | -0.15 | 0.05 | |
| V | hsCRP | Immediate Recall | 567 | adjusted | -0.02 | -0.12 | 0.08 | |
| V | IL-6 | Backwards Digit Span | 588 | unadjusted | -0.16 | -0.24 | -0.07 | |
| V | IL-6 | Backwards Digit Span | 588 | adjusted | -0.11 | -0.20 | -0.02 | |
| V | IL-6 | Delayed Recall | 588 | unadjusted | -0.15 | -0.24 | -0.05 | |
| V | IL-6 | Delayed Recall | 588 | adjusted | -0.09 | -0.18 | -0.01 | |
| V | IL-6 | Immediate Recall | 588 | unadjusted | -0.05 | -0.16 | 0.06 | |
| V | IL-6 | Immediate Recall | 588 | adjusted | -0.01 | -0.12 | 0.09 | |
| V | IL-10 | Backwards Digit Span | 588 | unadjusted | -0.06 | -0.15 | 0.04 | |
| V | IL-10 | Backwards Digit Span | 588 | adjusted | -0.05 | -0.15 | 0.04 | |
| V | IL-10 | Delayed Recall | 588 | unadjusted | 0.01 | -0.10 | 0.13 | |
| V | IL-10 | Delayed Recall | 588 | adjusted | 0.02 | -0.09 | 0.12 | |
| V | IL-10 | Immediate Recall | 588 | unadjusted | -0.03 | -0.14 | 0.08 | |
| V | IL-10 | Immediate Recall | 588 | adjusted | -0.02 | -0.13 | 0.09 | |
| V | IL-8 | Backwards Digit Span | 588 | unadjusted | -0.17 | -0.27 | -0.07 | |
| V | IL-8 | Backwards Digit Span | 588 | adjusted | -0.17 | -0.26 | -0.08 | |
| V | IL-8 | Delayed Recall | 588 | unadjusted | -0.17 | -0.29 | -0.06 | |
| V | IL-8 | Delayed Recall | 588 | adjusted | -0.17 | -0.27 | -0.06 | |
| V | IL-8 | Immediate Recall | 588 | unadjusted | -0.18 | -0.28 | -0.09 | |
| V | IL-8 | Immediate Recall | 588 | adjusted | -0.19 | -0.28 | -0.09 | |
| V | TNF-α | Backwards Digit Span | 588 | unadjusted | -0.1 | -0.21 | 0.01 | |
| V | TNF-α | Backwards Digit Span | 588 | adjusted | -0.1 | -0.23 | 0.02 | |
| V | TNF-α | Delayed Recall | 588 | unadjusted | -0.06 | -0.18 | 0.05 | |
| V | TNF-α | Delayed Recall | 588 | adjusted | -0.05 | -0.16 | 0.06 | |
| V | TNF-α | Immediate Recall | 588 | unadjusted | -0.08 | -0.18 | 0.02 | |
| V | TNF-α | Immediate Recall | 588 | adjusted | -0.07 | -0.18 | 0.04 | |
| V | IL-1β | Backwards Digit Span | 588 | unadjusted | -0.13 | -0.25 | -0.01 | |
| V | IL-1β | Backwards Digit Span | 588 | adjusted | -0.13 | -0.25 | -0.01 | |
| V | IL-1β | Delayed Recall | 588 | unadjusted | -0.18 | -0.31 | -0.05 | |
| V | IL-1β | Delayed Recall | 588 | adjusted | -0.17 | -0.29 | -0.05 | |
| V | IL-1β | Immediate Recall | 588 | unadjusted | -0.11 | -0.22 | 0.00 | |
| V | IL-1β | Immediate Recall | 588 | adjusted | -0.11 | -0.21 | -0.02 | |

Wave IV was conducted in 2008 when participants had a median age of 28 years (IQR: 26-29 years), Wave V was conducted in 2016-2018 when participants had a median age of 38 (IQR: 36-39 years).

CAIDE=Cardiovascular Risk Factors, Aging, and Incidence of Dementia, ATN=Amyloid (A), tau (T), and neurodegeneration (N)

APOE Status=Apolipoprotein E, defined as having at least one ε4 allele (i.e. those with APOE ε2/ε4, ε3/ε4, or ε4/ε4 phenotypes vs. ε2/ε2, ε3/ε3, or ε2/ε3).

CAIDE score version 1 (v1) is a weighted sum of education, sex, age, total cholesterol, systolic blood pressure, body mass index, and physical activity. Version 2 (v2) includes the same variables and adds APOE status. APOE status defined as having at least one ε4 allele (i.e. those with APOE ε2/ε4, ε3/ε4, or ε4/ε4 phenotypes vs. ε2/ε2, ε3/ε3, or ε2/ε3).

hsCRP=high sensitivity C-Reactive Protein, TNF-α=Tumor Necrosis Factor alpha, IL=Interleukin, NfL=Neurofilament Light

#### Table S4. Associations Presented in Figure S3.

| **Wave** | **Independent Variable** | **Cognitive Domain** | **N** | **Model** | **Beta Estimate** | **95% CI** | |
| --- | --- | --- | --- | --- | --- | --- | --- |
| **CAIDE Scores** | |  |  |  |  |  |  |
| IV | CAIDE v1 | Backward Digit Span | 412 | unadjusted | -0.06 | -0.10 | -0.02 |
| IV | CAIDE v1 | Backward Digit Span | 412 | adjusted | -0.05 | -0.09 | -0.01 |
| V | CAIDE v1 | Backward Digit Span | 412 | unadjusted | -0.03 | -0.08 | 0.03 |
| V | CAIDE v1 | Backward Digit Span | 412 | adjusted | -0.01 | -0.07 | 0.04 |
| IV | CAIDE v1 | Delayed Recall | 412 | unadjusted | -0.05 | -0.10 | 0.00 |
| IV | CAIDE v1 | Delayed Recall | 412 | adjusted | -0.03 | -0.08 | 0.01 |
| V | CAIDE v1 | Delayed Recall | 412 | unadjusted | -0.02 | -0.07 | 0.02 |
| V | CAIDE v1 | Delayed Recall | 412 | adjusted | -0.01 | -0.06 | 0.03 |
| IV | CAIDE v1 | Immediate Recall | 412 | unadjusted | -0.05 | -0.09 | -0.01 |
| IV | CAIDE v1 | Immediate Recall | 412 | adjusted | -0.04 | -0.08 | 0.00 |
| V | CAIDE v1 | Immediate Recall | 412 | unadjusted | -0.01 | -0.05 | 0.03 |
| V | CAIDE v1 | Immediate Recall | 412 | adjusted | -0.01 | -0.04 | 0.03 |
| IV | CAIDE v2 | Backward Digit Span | 406 | unadjusted | -0.06 | -0.10 | -0.03 |
| IV | CAIDE v2 | Backward Digit Span | 406 | adjusted | -0.05 | -0.08 | -0.01 |
| V | CAIDE v2 | Backward Digit Span | 406 | unadjusted | -0.04 | -0.09 | 0.01 |
| V | CAIDE v2 | Backward Digit Span | 406 | adjusted | -0.03 | -0.08 | 0.03 |
| IV | CAIDE v2 | Delayed Recall | 406 | unadjusted | -0.06 | -0.10 | -0.02 |
| IV | CAIDE v2 | Delayed Recall | 406 | adjusted | -0.04 | -0.08 | 0.00 |
| V | CAIDE v2 | Delayed Recall | 406 | unadjusted | -0.03 | -0.07 | 0.01 |
| V | CAIDE v2 | Delayed Recall | 406 | adjusted | -0.02 | -0.06 | 0.03 |
| IV | CAIDE v2 | Immediate Recall | 406 | unadjusted | -0.06 | -0.11 | -0.02 |
| IV | CAIDE v2 | Immediate Recall | 406 | adjusted | -0.05 | -0.09 | -0.01 |
| V | CAIDE v2 | Immediate Recall | 406 | unadjusted | -0.02 | -0.05 | 0.02 |
| V | CAIDE v2 | Immediate Recall | 406 | adjusted | -0.01 | -0.05 | 0.02 |
| **APOE ε4 Status** | |  |  |  |  |  |  |
| IV | e4 carrier | Backward Digit Span | 1063 | unadjusted | -0.11 | -0.30 | 0.08 |
| IV | e4 carrier | Backward Digit Span | 1063 | adjusted | -0.12 | -0.30 | 0.07 |
| V | e4 carrier | Backward Digit Span | 1063 | unadjusted | 0.00 | -0.17 | 0.17 |
| V | e4 carrier | Backward Digit Span | 1063 | adjusted | -0.01 | -0.18 | 0.16 |
| IV | e4 carrier | Delayed Recall | 1063 | unadjusted | -0.08 | -0.25 | 0.08 |
| IV | e4 carrier | Delayed Recall | 1063 | adjusted | -0.08 | -0.24 | 0.08 |
| V | e4 carrier | Delayed Recall | 1063 | unadjusted | -0.05 | -0.27 | 0.18 |
| V | e4 carrier | Delayed Recall | 1063 | adjusted | -0.04 | -0.26 | 0.18 |
| IV | e4 carrier | Immediate Recall | 1063 | unadjusted | -0.02 | -0.19 | 0.15 |
| IV | e4 carrier | Immediate Recall | 1063 | adjusted | -0.02 | -0.18 | 0.15 |
| V | e4 carrier | Immediate Recall | 1063 | unadjusted | 0.07 | -0.10 | 0.24 |
| V | e4 carrier | Immediate Recall | 1063 | adjusted | 0.08 | -0.09 | 0.24 |

Wave IV was conducted in 2008 when participants had a median age of 28 years (IQR: 26-29 years), Wave V was conducted in 2016-2018 when participants had a median age of 38 (IQR: 36-39 years).

CAIDE=Cardiovascular Risk Factors, Aging, and Incidence of Dementia, ATN=Amyloid (A), tau (T), and neurodegeneration (N)

APOE Status=Apolipoprotein E, defined as having at least one ε4 allele (i.e. those with APOE ε2/ε4, ε3/ε4, or ε4/ε4 phenotypes vs. ε2/ε2, ε3/ε3, or ε2/ε3).

CAIDE score version 1 (v1) is a weighted sum of education, sex, age, total cholesterol, systolic blood pressure, body mass index, and physical activity. Version 2 (v2) includes the same variables and adds APOE status. APOE status defined as having at least one ε4 allele (i.e. those with APOE ε2/ε4, ε3/ε4, or ε4/ε4 phenotypes vs. ε2/ε2, ε3/ε3, or ε2/ε3).

# References

1. McDade TW, Miller A, Tran TT, et al. A highly sensitive multiplex immunoassay for inflammatory cytokines in dried blood spots. *American Journal of Human Biology* 2021; 33: e23558.

2. Schmeer KK, Tarrence J. Racial-ethnic Disparities in Inflammation: Evidence of Weathering in Childhood? *J Health Soc Behav* 2018; 59: 411–428.

3. Momkus J, Aiello AE, Stebbins R, et al. Sociodemographic patterns in biomarkers of aging in the Add Health cohort. *Biodemography and Social Biology* 2024; 69: 57–74.

4. Noppert GA, Stebbins RC, Dowd JB, et al. Socioeconomic and race/ethnic differences in immunosenescence: Evidence from the Health and Retirement Study. *Brain, Behavior, and Immunity* 2023; 107: 361–368.

5. Javed Z, Haisum Maqsood M, Yahya T, et al. Race, Racism, and Cardiovascular Health: Applying a Social Determinants of Health Framework to Racial/Ethnic Disparities in Cardiovascular Disease. *Circulation: Cardiovascular Quality and Outcomes* 2022; 15: e007917.

6. Pool LR, Ning H, Lloyd‐Jones DM, et al. Trends in Racial/Ethnic Disparities in Cardiovascular Health Among US Adults From 1999–2012. *Journal of the American Heart Association*; 6: e006027.

7. Barnes LL, Bennett DA. Alzheimer’s Disease In African Americans: Risk Factors And Challenges For The Future. *Health Affairs* 2014; 33: 580–586.

8. Matthews KA, Xu W, Gaglioti AH, et al. Racial and ethnic estimates of Alzheimer’s disease and related dementias in the United States (2015–2060) in adults aged ≥65 years. *Alzheimer’s & Dementia* 2019; 15: 17–24.

9. Williams DR, Lawrence JA, Davis BA. Racism and Health: Evidence and Needed Research. *Annu Rev Public Health* 2019; 40: 105–125.

10. Williams DR, Mohammed SA. Racism and Health I: Pathways and Scientific Evidence. *American Behavioral Scientist* 2013; 57: 1152–1173.
